# Human pluripotent stem cell (hPSC) and organoid models of autism: opportunities and limitations

**DOI:** 10.1038/s41398-023-02510-6

**Published:** 2023-06-21

**Authors:** Savannah Kilpatrick, Courtney Irwin, Karun K. Singh

**Affiliations:** 1 Donald K. Johnson Eye Institute, Krembil Research Institute, University Health Network, Toronto, ON Canada; 2 Department of Biochemistry and Biomedical Science, McMaster University, Hamilton, ON Canada; 3 Department of Laboratory Medicine and Pathobiology, Faculty of Medicine, University of Toronto, Toronto, ON Canada; 4 Department of Ophthalmology and Vision Sciences, University of Toronto, Toronto, ON Canada

**Keywords:** Autism spectrum disorders, Stem cells

## Abstract

Autism spectrum disorder (ASD) is a complex neurodevelopmental disorder caused by genetic or environmental perturbations during early development. Diagnoses are dependent on the identification of behavioral abnormalities that likely emerge well after the disorder is established, leaving critical developmental windows uncharacterized. This is further complicated by the incredible clinical and genetic heterogeneity of the disorder that is not captured in most mammalian models. In recent years, advancements in stem cell technology have created the opportunity to model ASD in a human context through the use of pluripotent stem cells (hPSCs), which can be used to generate 2D cellular models as well as 3D unguided- and region-specific neural organoids. These models produce profoundly intricate systems, capable of modeling the developing brain spatiotemporally to reproduce key developmental milestones throughout early development. When complemented with multi-omics, genome editing, and electrophysiology analysis, they can be used as a powerful tool to profile the neurobiological mechanisms underlying this complex disorder. In this review, we will explore the recent advancements in hPSC-based modeling, discuss present and future applications of the model to ASD research, and finally consider the limitations and future directions within the field to make this system more robust and broadly applicable.

## ASD overview and 2D/3D modeling

Autism spectrum disorder (ASD) is a highly prevalent neurodevelopmental disorder (NDD) that impacts as many as 1/44 of children in the United States [1]. Clinical presentations of ASD vary widely among individuals but must include repetitive, restricted behaviors and social deficits [2]. To add to this complexity, comorbidities often include epilepsy and seizure disorders (up to 30%), intellectual disability (>30%), ADHD, gastrointestinal disorders (up to 70%), anxiety, and depression [3–9]. Risk factors for ASD can occur prenatally, perinatally, and postnatally and include genetic disruptions and environmental insults, of which there is likely a combinatorial or synergistic effect.

Twin studies were one of the first indicators of the genetic component to ASD, and while heritability estimates can range from 45 to 90%, it is more broadly thought to be 70–80% [10, 11]. More recently, cohort-based sequencing studies have provided a genetic framework to studying ASD and have identified several hundred implicated genes. Genetic disruptions include inherited rare variants and less common de novo mutations that exist as single nucleotide polymorphisms (SNPs), copy number variants (CNVs), and chromosomal abnormalities [12–19] Despite the immense progress in identifying ASD-risk genes, the encoded proteins and resulting pathobiology remains elusive. Scientists have turned to genetic modeling to better understand the molecular, cellular, and functional (circuit-based) consequences to disruption in these ASD-risk genes [20].

Genetic models of ASD have most commonly included mouse and human cell lines, which provide biologically and clinically relevant opportunities for study [21, 22]. These models are not without their caveats, however, as the development of a mouse brain excludes human-specific processes such as brain gyrification, the protracted development and formation of particular neural cell types, and human-specific gene transcriptional programs. In addition, mouse behavioral assays are often not translatable to the complex clinical presentations of those with ASD (reviewed in refs. [23, 24]). Conversely, human cell lines produce reliable, replicable environments for testing simple pathways, but are reductive and lack the ability to mimic complex developmental brain processes. Further, they can lack specific cell types and structures that play a crucial role in development, such as brain vascularization.

Induced pluripotent stem cells (iPSCs), which can be generated from human blood or skin-derived fibroblasts, have transformed the use of human cellular models [25]. iPSCs retain the unique genetic background from the individual, which is important given that idiopathic ASD represents roughly 80–85% of the ASD population [26]. Through directed differentiation, iPSCs can produce any germ layer cell types to model complex and inaccessible tissue such as the developing brain, allowing for a putatively unlimited supply of patient-specific tissues to study disease processes or drug screening [27] (reviewed in ref. [28], summarized in Table 1).

**Table 1:** Summary of current ASD model classes with relevant advantages and disadvantages.

Model	Application(s)	Advantage	Disadvantage
Mouse models	In vitro, in vivo studies	Whole-body system capable of undergoing core developmental milestones shared between species	Failure to recapitulate species-specific cell types, gene expression, and developmental trajectories (ex: protracted maturation in humans)
Postmortem tissues	Cellular characterizations	Directly sourced patient samples that were previously involved in a whole-body system	Samples subject to degradation, timepoints may be irrelevant to the pathology of interest
Clinical imaging data (fMRI, MRI)	Imaging of whole-system in real time	Patient-specific data that can be performed longitudinally	Low resolution, and longitudinal studies often fail to sample a consistent population across time
Clinical functional studies (EEG)	Recording of whole-system in real time	Patient-specific data that can be performed longitudinally	Low resolution, and longitudinal studies often fail to sample a consistent population across time
Peripheric tissue (blood)	In vitro/molecular studies	Patient-specific data that can be drawn longitudinally	Data is generalizable and not neural-specific
Human cell lines (SH-SY5Y, HEK239, etc.)	In vitro studies	Replicable, scalable model, amendable to CRISPR editing	Differentiation protocols fail to generate relevant cell types
Behavioral studies	Human and mouse behavior phenotyping	Direct assessment of core ASD criteria (restricted interests, repetitive tasks)	Failure to address underlying pathology on a biological or molecular level
Computational studies	In silico	Non-invasive, scalable	Requires training and use of datasets that are currently poorly understood
Directed differentiation (2D) of iPSCs	In vitro modeling	Quick, scalable production of relevant cell types (NPCs, neurons) from patient source or isogenic background	Reductive, with exclusion of a 3D Cellular microenvironment
Organoids	3D in vitro modeling	Relevant, diverse cell types from patient source or isogenic background	Lack of vascularization, and a higher degree of heterogeneity

*ASD* autism spectrum disorder, (*f)MRI* (functional) magnetic resonance imaging, *EEG* electroencephalogram, *NPC* neural progenitor cell.

An essential process during brain development is the genesis and differentiation of neural stem cells (neurogenesis), which can be captured using hPSCs. Neurogenesis describes the emergence of various differentiated brain cell types from neural stem cells and neural progenitor cells (NPCs). Both precursor cell types are important for the formation of the neurons and glia that populate the cerebral cortex, and can be classified based on their mitotic state, location, and polarity (apical or basal). The polarity of an NPC or neural stem cell reflects the positioning of essential proteins and organelles such as the Golgi apparatus and can influence the cell fate and diversity of daughter cells. Disruptions in cell polarity have been associated with a number of NDDs including Fragile X, SCZ, and ASD [29–32].

NPCs differ from neural stem cells in that their pluripotent fate is more restricted; they have limited proliferation and are capable of producing most neural and glial cell types in the CNS. Given the frequent presentations of macrocephaly amongst ASD individuals, it is possible that excessive neural growth is an underlying factor that may contribute towards ASD pathogenesis, which has been examined using hPSC-derived NPCs [33–36].

NPC proliferation has been characterized using patient-derived hPSCs and can even be used to stratify subpopulations amongst patients with ASD. In a recent study, hPSCs derived from an ASD cohort that had either idiopathic ASD or a 16p11.2 microdeletion were used to generate NPCs to examine proliferating pathways. The team found that hPSCs derived from macrocephalic individuals with either the 16p11.2 deletion or idiopathic ASD showed increased proliferation and DNA synthesis and proliferation, whereas the remaining probands displayed the opposite trend [37]. The lines were categorized as hyperproliferative and hypoproliferative NPCs and were then treated with basic fibroblast growth factor (bFGF), a mitogen response element that can prime cells for cortical progenitor proliferation. Interestingly, NPCs from the hyperproliferative group displayed a blunted response to bFGF, whereas the hypoproliferative group showed an increase in DNA synthesis sensitivity and response to stimulation. This work highlights the complexities of disease modeling and how patient-derived NPCs can be used to identify subpopulations amongst heterogeneous clinical datasets.

hPSC-derived NPCs enable researchers to examine complex biological processes relating to proliferation and neuronal differentiation. Directed differentiation can produce robust cultures that can be subjected to high-throughput screening, drug testing and phenotyping [38]. Terminal differentiation can be achieved through the addition of various compounds and transcription factors to broaden the window of development that is examined [39–42], an example being the Ngn2 system that produces glutamatergic-like excitatory neurons. Two major caveats of this system are the reductive and overly simplistic 2D nature of the cultures, and their short lifespans. Unlike hPSCs, NPCs can only be passaged a discreet number of times, limiting the scalability of the model.

**Fig. 1:**
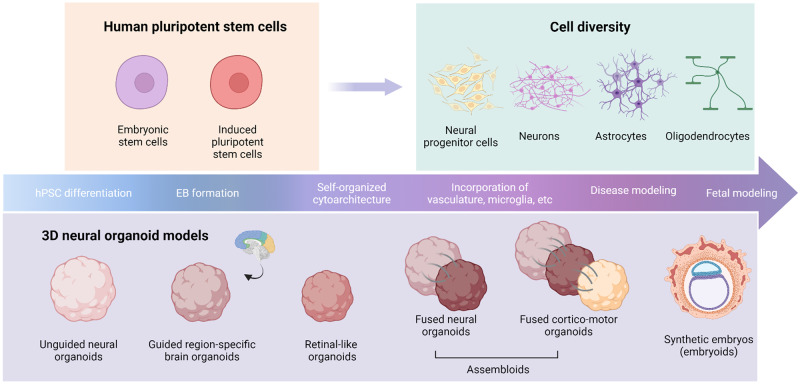
2D and 3D Neural modeling opportunities from hPSC cultures. Schematic representation of human pluripotent stem cell (hPSC) differentiation into neural organoids. The cell-type diversity is highlighted in the right panel, and the organoid and assembloid opportunities are shown below.

Although hPSC-derived monolayer cultures have deepened our understanding of CNS development, function, and pathology, 2D spatial-organizational constraints limit their ability to model three-dimensional (3D) tissue architecture with complex cell-cell and cell-extracellular matrix (ECM) interactions [43, 44]. Advances in stem cell technology over the past decade have led to the emergence of 3D self-organizing brain organoid models that recapitulate key cellular, structural, and circuital features of human development and disease (summarized in Table 2) [45]. These models were first pioneered with the use of hPSCs by Dr. Yoshiki Sasai’s group with the generation of cortical tissues and 3D optic cup structures in the early 2000s, which has since broadened towards established protocols that exploit either intrinsic or extrinsic signaling pathways to coax the differentiation of hPSCs toward cellular lineages reminiscent of whole or region-specific brain development, respectively [46] (Fig. 1).

**Table 2:** Overview of hPSC-derived ASD genetic modeling.

Genetic variant type	Model system (guided vs unguided, 2D vs 3D)	Genetic variant	References	Phenotyping	Unique phenotypes/findings	Summary
CNV	Guided forebrain and dissociated cultures	22q11.2 DEL	Khan et al. [148]	Bulk/single-cell transcriptomics, calcium imaging, CRISPR/Cas9-mediated gene editing, ICC	Rescue of abnormal calcium phenotype with antipsychotic	Transcriptional profiling and phenotyping across 100 days showed disruptions in neuronal excitability genes and calcium imaging
	Guided corticostriatal assembloids	22q13.3 DEL	Miura et al. [62]	Single-cell transcriptomics, optogenetics, calcium imaging	Hyperactivity of medium spiny neurons was only apparent in assembloid model	Defects in corticostriatal connectivity and abnormal calcium signaling
	Guided cortical organoids	15q13.3 DUP	Meganathan et al. [209]	Bulk transcriptomics, neurite outgrowth assays, ICC	Pharmacological rescue of neuron migration and ER stress	Elevated endoplasmic reticulum stress, increased NPC proliferation, disrupted Wnt and axon guidance signaling
	Guided cortical organoids	16p11.2 DEL & DUP	Urresti et al. [111]	Bulk transcriptomics, proteomics, cellular and molecular studies	Gene-dosage-dependent changes in organoid size in 16p11.2 duplication and deletion lines	Micro- and macrocephaly associated with duplication and deletion, respectively, dysregulated neuronal maturation, migration, and synaptic processes
	Guided cortical organoids and dissociated cultures	7q11.23 DEL & DUP	Mihailovich et al. [210]	Ribosomal profiling, transcriptomics, proteomics,	Identified the REST pathway as a key mediator to 7q11.23 duplication	Gene-dosage effects on neuron excitability and differentiation in 7q11.23 DEL and DUP patients
	Guided forebrain organoids	17p13.3 DEL	Iefremova et al. [211]	Molecular assays, gene rescue, drug rescue, ICC	Rescue of abnormal morphology through Wnt signaling	Disruptions in ventricular zone architecture and premature neuronal differentiation
	NPCs and cortical neurons	7q11.23 DEL	Chailangkarn et al. [19]	Bulk transcriptomics, calcium imaging, MEA, electrophysiology, neuronal morphology studies	Established the role of a single gene within the CNV that drives cellular phenotypes and viability in NPCs	Reduced viability in 7q11.23 DEL NPCs, and excessive dendritic growth in both iPSC- and postmortem- derived neurons
	NGN2-induced neurons	7q11.23 DUP	Cavallo et al. [212]	Drug screening, molecular studies	Performed a high-throughput drug screen from a library of 1000 compounds on patient-derived 2D glutamatergic neurons	Identified multiple HDAC-inhibitor compounds that were capable of rescuing expression of a prominent 17q11.2 driver gene
	NGN2-induced neurons	15q13.3 DEL	Unda et al. [18]	MEA, Electrophysiology, cellular morphology	Characterizing the proteomic network of CNV driver genes in a model of ASD	15q13.3 DEL NGN2 neurons display reduced synaptic maturation and altered AIS phenotypes in an OTUD7A-dependent manner
	NPC and cortical neurons cultures	Xp11.2 DEL	Ross et al. [213]	Electrophysiology, molecular studies, CRISPR editing	Examined the effects of locus deletions on neural circuitry	Reduced miniature excitatory postsynaptic current frequency and NMDA receptor function
	Unguided neural organoids	7q11.2 DEL	Wegscheid et al. [214]	Bulk transcriptomics, ICC, molecular assays	Identified Ras-depending increases in NPC proliferation	7q11.2 DEL patient-derived organoids show increased NPC proliferation, disrupted neural differentiation, and increased neural death
	Unguided neural organoids	17p13.3 DEL	Bershteyn et al. [215]	Single-cell transcriptomics, time-lapse imaging, ICC	Identified mitotic defects in human-specific outer radial glia populations	Reduced migration of cells during neurite outgrowth, increased apoptosis of cells lining VZ-like rosettes
Single gene KO or SNP	Guided forebrain organoids	CNTNAP2	de Jong et al. [216]	Single-cell and bulk transcriptomics, CRISPR Correction, light sheet microscopy, ICC	CRISPR Correction was capable of rescuing both morphological and transcriptomic alterations in patient-derived lines	CNTNAP2 is predominantly expressed in several excitatory neuron subpopulations, and leads to cortical overgrowth
	Guided cortical organoids	DISC1	Qian et al. [217]	ICC, cellular assays	Study improved nutrient flow and lamination by cutting cortical organoids into thick organotypic organoid slices	Laminar disruptions and deficits in cortical neuron fate
	Guided forebrain organoids	FMRP	Raj et al. [218]	Bulk transcriptomics, protein translation assays, flow cytometry, ICC	PI3K pathway was identified as a key regulator to the abnormal protein translation	Fragile X-Derived NPCs show global increases in protein translation
	Guided cortical organoids	MECP2	Trujillo et al. [219]	MEA, drug screening, CRISPR/Cas9-mediated gene editing	Drug screen in cortical organoids showed increased gene expression in neurotransmitter markers	Partial, but not the complete rescue of network activity was achieved with the use of two pharmacological compounds
	Guided cortical organoids	PTEN	Pigoni et al. [220]	Single-cell and spatial transcriptomics, proteomics	Despite differences in early phenotypes, all lines showed consistent disruptions in the signaling of local circuits	Defects in outer radial glia progenitors and deep-layer projection neurons are influenced by the patient background
	Guided cortical organoids	SUV420H1, ARID1B and CHD8	Paulsen et al. [221]	Single-cell transcriptomics, snATAC-Seq, MEA, Calcium imaging, proteomics	GABAergic interneurons and deep-layer projection neurons were identified as the vulnerable cell type. Divergence was mainly identified in the molecular targets of the risk genes	Identified phenotypic convergence of ASD-risk gene-derived organoids on asynchronous neuronal development
	Guided cortical organoids	TCF4	Papes et al. [222]	MEA, scRNA Seq, CRISPR/Cas9-mediated gene editing and correction, drug rescue, ICC	TCF4-mediated Wnt signaling disruptions lead to reduced expression of *SOX* genes	Reduced NPC proliferation and impaired neuronal differentiation
	Guided cortical organoids	UBE3A	Sun et al. [223]	Electrophysiology, drug rescue, and molecular and mouse model studies	Treatment with potassium channel antagonist rescued seizure phenotype in both mouse and human models	Hyperexcitability phenotype in addition to increased synchronous firing
	Unguided neural organoids	UBE3A	Sen et al. [224]	Calcium imaging, drug rescue, ICC	Paternal UBE3A was shown to be silenced in early-stage cerebral organoids	Aberrant localization of UBE3A in mutant cerebral organoids that is partially rescued by topoisomerase inhibitors
	Unguided forebrain assembloids	CACNA1C	Birey et al. [57]; Birey et al. [81]	Bulk transcriptomics, live cell imaging, calcium imaging	Novel protocol for the formation of assembloids	Irregular interneuron migration saltation which was partially rescued by an L-type calcium channel blocker and GABA receptor antagonist
	Unguided neural organoids	CHD8	Villa et al. [225]	Single-cell transcriptomics, CRISPR/Cas9-mediated gene editing, ICC	CHD8 haploinsufficiency models display impaired neurodevelopmental trajectories with accelerated inhibitory neuron development and impaired excitatory neuron development	Macrocephalic presentations and altered mRNA processing in mature neurons
	Unguided neural organoids	CHD8	Wang et al. [226]	Bulk transcriptomics, CRISPR/Cas9-mediated gene editing	Found convergence of DEGs and pathways dysregulated in ASD, bipolar disorder, and schizophrenia	Dysregulation in genes involved in neuronal migration
	Unguided neural organoids	MECP2	Mellios et al. [227]	Electroporation, sh-mediated knockdown, microRNA profiling, mouse in vivo studies	MECP2 regulates several miRNAs that are candidates for rescuing disease pathology	MECP2 regulates several miRNAs involved in early human neurogenesis, and KO models display alterations in AKT/ERK signaling
	Unguided neural organoids	RAB39b	Zhang et al. [228]	CRISPR/Cas9-mediated gene editing	RAB39b operates through PI3K-AKT-mTOR signaling	Mutations in RAB39b result in macrocephalic-phenotypes and hyperproliferative NPCs with defective differentiation capacities
	Unguided neural organoids	CDK5RAP2	Lancaster et al. [49]	ICC, gene rescue, molecular studies	Proof of principal in modeling microcephalic disorders that are often ASD- associated	Disrupted symmetrical: asymmetrical division in patient-derived cerebral organoids
	Unguided neural organoids	ACTL6B	Wenderski et al. [229]	Bulk transcriptomics, ATAC-Seq, exome sequencing, molecular assays, mouse studies	Insight into chromatin regulation in a model of ASD	Alterations in activity-dependent transcription
	Unguided neural organoids	SHANK3	Malara et al. [230]	Electron microscopy, ICC, molecular assays	Rare cell type identified in cerebral organoids shows promise to study disorders of CNS myelination in models of ASD	Alterations in myelin-producing cells
	Unguided neural organoids	SHANK3	Wang et al. [231]	Single-cell transcriptomics, electrophysiology, CRISPR/Cas9-mediated gene editing, ICC	Protocol included formation of neural organoids from single rosettes, SHANK3 model showed impaired RhoA Signaling	Deficits in intrinsic excitability and reductions in several clustered protocadherins
Idiopathic	Unguided neural organoids	N/A	Mariani et al. [232]	Bulk transcriptomics, electrophysiology, ICC	Recapitulated the phenotype with RNAi of FOXG1	FOXG1 overexpression causes an increase in inhibitory neuron formation found amongst patients with idiopathic autism
	Unguided neural organoids	N/A	Ilieva et al. [164]	Proteomics, metabolic studies, ICC	Identified potential biomarkers and metabolic deficiencies in cerebral organoids derived from individuals with idiopathic autism	Reduced glutamate release and spontaneous firing rate in neurons
	Astrocyte and mature neural co-cultures	N/A	Russo et al. [106]	MEA phenotyping, molecular studies	ASD-derived astrocytes hindered the maturation of neurons derived from control lines	Reduced synaptic and glutamatergic activity in neurons. Morphologic and synaptogenesis impairments were rescued by co-culture with control astrocytes lines

*ASD* autism spectrum disorder, *ICC* immunocytochemistry, *NPC* neural progenitor cell, *MEA* multielectrode array, *SEM* scanning electron microscopy, *snATC Seq* single-nucleus assay for transposase-accessible chromatin.

Unguided approaches rely on the spontaneous differentiation of hPSCs to ECM-embedded heterogeneous cerebral tissue [47]. The resultant unguided neural organoids (UNOs), formerly known as cerebral organoids [48], exhibit discrete regionalization reminiscent of in vivo human whole-brain development, such as markers of forebrain, midbrain, hindbrain, dorsal cortex, prefrontal cortex, hippocampal, occipital lobe, ventral forebrain, choroid plexus, meningeal, and retinal identity [47, 49]. Furthermore, they demonstrate cellular and structural features unique to human cortical progenitor zone organization, such as layers resemblant of ventricular and subventricular zones [49]. Epigenomic and single-cell transcriptomic analyses of UNO tissue have revealed a remarkable similarity to the early developing fetal cortex [50, 51]. However, undirected hPSC differentiation inherently results in stochastic organoid cellular composition that can hinders batch reproducibility [49]. Alternatively, guided organoid approaches incorporate exogenous signaling factors to direct hPSC differentiation towards region-specific lineages, such as those present in the cerebral cortex [52–55], forebrain [56–59], medial ganglionic eminence [54], midbrain [56, 60], thalamus [61], striatum [62], pituitary [63], hypothalamus [56, 64, 65], choroid plexus [66], cerebellum [67], brainstem [68], and spinal cord [69, 70]. These organoids generally display less batch-to-batch heterogeneity than their undirected counterparts and therefore may be more conducive to quantitative analyses [46]. Guided organoids have even been combined to generate assembloids comprised of different brain regions, which provide incredible promise to study pathophysiology within affected circuits.

## Brain structure, assembloids, and circuitry

Broad structural and circuit abnormalities have been identified in multiple brain regions of individuals with ASD. In addition to generalized macrocephaly, ASD brains can have structural abnormalities within areas of higher order cognitive processing such as the cerebellum, frontal lobe, and limbic system [71, 72], and even manifest in enlargement of the ventricular cavities where newborn neurons originate [73, 74]. Due to a lack of standardized clinical imaging and EEG recordings, it is impossible to know how pervasive these brain abnormalities are within ASD populations alone, but recent population studies have estimated a frequency of 30–50% [75]. These structural changes are often subtle and variable across individuals with ASD, suggesting that dysregulated circuitry between affected regions and altered molecular pathways may be the underlying cause to this presentation [76–78].

Unguided neural organoids (UNOs) have been used to model both microcephaly and macrocephaly in disease contexts [49, 56]. A primary example includes studies of *PTEN* variants in ASD populations that are comorbid for macrocephaly [79]. Loss of PTEN function was investigated in UNOs by use of isogenic hPSC homozygous mutant lines; concordant with the loss of function mutations found in NDD populations with macrocephaly, the UNOs displayed an increase in size across development, in addition to aberrant tissue folding identified through light sheet microscopy [79]. More recently, PTEN gene-dosage sensitivity was assessed by comparing the isogenic KO to a lentiviral overexpression hPSC line, to model the 10q23.31 microduplication associated with patients with autosomal dominant primary microcephaly. Here it was found that UNO size was inversely proportional to PTEN expression, and could be rescued by an AKT inhibitor that acts on a known PTEN pathway [80]. This demonstrates the use of UNOs to model whole-brain structural abnormalities in ASD populations, and how they can be mined for pharmacological rescue.

Due to the developmental nature of the disorder, as well as the multiple brain regions affected, ASD may arise from miswiring amongst neural circuits during fetal development, with an enrichment in the developing cortex. Advances in guided neural organoids (GNOs, or brain-region-specific organoids) have enabled investigation into how different areas of the brain interact in a disease model. When merged, the resulting assembloids provide the necessary environment for cell-cell interactions and complex developmental processes including integration into circuitry. Assembloids can be examined to assess gross structural abnormalities, the migration of neuronal subpopulations, as well as inter-assembloid circuitry. Assembloids have included the combination of cortical (dorsal) and ventral forebrain organoids [58], cortical-thalamus [61], cortical-striatum [62], cortical-subpial spheroids [81], and even tri-part assembloids consisting of cortical-spinal fused to skeletal muscle assembloids [70]. These new model systems allow for the de novo generation of synthetic circuits in the lab, which have been shown to generate spontaneous neural oscillations comparable to that of the developing human brain [82].

Migration and circuit-based disruptions have been described in multiple ASD models, and in patients are often identified through MRI of gross structural abnormalities in the brain or inferred from EEG recordings of epileptic or paroxysmal activity [75, 83, 84] (reviewed in refs. [85–87]). EEG abnormalities include an increased frequency of focal spikes, or localized activity to a particular area of the brain [84, 88, 89]. Several wavelength frequency abnormalities have been characterized within ASD cohorts, including an increase in low-frequency (delta and theta) and high frequency (beta and gamma) wavelengths which is contrasted by a reduction in mid-range alpha frequencies, producing a characteristic U-shaped electrophysiological profile, in which the extremities of the power spectrum are enhanced in ASD populations and the mid-range values are reduced. Organoids generate many of the neural stem cell populations and mature cell types in the brain, and are capable of producing many of the EEG wavelengths mentioned above, in addition to increased firing rate, burst frequency, synchronicity, and population spiking across several months of development [45]. These qualities make organoids a promising model to examine functional aspects of ASD in a developing model. Assembloid systems are likely capable of complex neural activity and ossciations [90], and importantly can be used to probe for the innervation and migration of specific cell types in order to assess cellular circuitry between distinct brain regions.

Due to the nature of these tools and an inability to examine ASD pathology at a cellular resolution, the causative cell populations remain unknown. One potential cell population that may drive these global abnormalities are GABAergic interneurons, which are known to regulate the power of upper and lower- frequencies in the developing brain [91]. It is possible that disruptions in the connectivity of these and other cell types in the fetal brain are what produce the epileptiform changes, which can occur through local miswiring or the failure of a cell population to migrate to its intended destination. It should be noted that these processes arise in early fetal development and occur well before the postnatal time point of clinical assessments such as MRI and EEG in ASD populations.

Neuronal migration is an essential process in the developing brain, where excitatory cells emerge from the ventricular zone to create laminar structures in a well-defined, spatiotemporal manner. Recently, it has been found that a subpopulation of inhibitory neurons is also born from cortical progenitor cells, a phenomenon that appears to be human-specific [92, 93]. The remaining inhibitory neurons follow later in development to emerge from proliferative zones in the ventral telencephalon to migrate into the cortex [94–96]. This migratory process is well characterized in the human brain and known to be disrupted in NDDs such as ASD, Tourette Syndrome, and epilepsy [97]. Interneuron migration was recently investigated in human 3D organoid models of Timothy syndrome (TS), a severe neurodevelopmental disorder caused by mutations in the calcium channel, *LTCC*. Using patient-derived forebrain assembloids composed of cortical (dorsal) and subpallium (ventral) organoids, the researchers were able to identify disruptions in GABAergic interneuron migration originating from the ventral organoid; specifically, their saltatory movements were more frequent but less efficient, moving a lesser distance than control lines. Abnormal calcium signaling was thought to underlie the migration defects, and targeted pharmacological activation of the mutated calcium channel was found to rescue the migration phenotype. Importantly, this abnormality was found exclusively in assembloid-derived ventral organoids, and not in ventral organoids alone, demonstrating the utility of this system in modeling complex circuitry.

Circuitry-based disruptions have also been described more broadly in copy number variant (CNV) models of ASD (15q11.3, 15q13.3, 22q11.2, 22q13.3, 1q21.1) [98–102], which frequently include epileptic comorbidities. These functional deficits in ASD have been explained as an imbalance in the ratio of excitatory: inhibitory cells and have been explored in a cohort of NDD patients with the known ASD-related CNV, 22q13.3. Importantly, this deletion encompasses a lead ASD-risk gene, *SHANK3*, which is highly expressed in human striatal tissue and has been implicated in corticostriatal circuitry disruptions in ASD individuals [62, 103]. Using an assembloid model of cortical organoids fused to striatal organoids, the group examined the axonal innervation from glutamatergic excitatory neurons into the striatum, which functionally connected to medium spiny neurons in the striatum organoid, similar to a developing brain [62]. Patient-derived assembloids were sliced or dissociated for single-cell patch clamp and calcium signaling, respectively, and both assays showed a hyperexcitable phenotype. Interestingly, this change was not present in individual striatal organoids, demonstrating the importance of assembloid modeling to capture complex circuit-based abnormalities in ASD models.

**Fig. 2:**
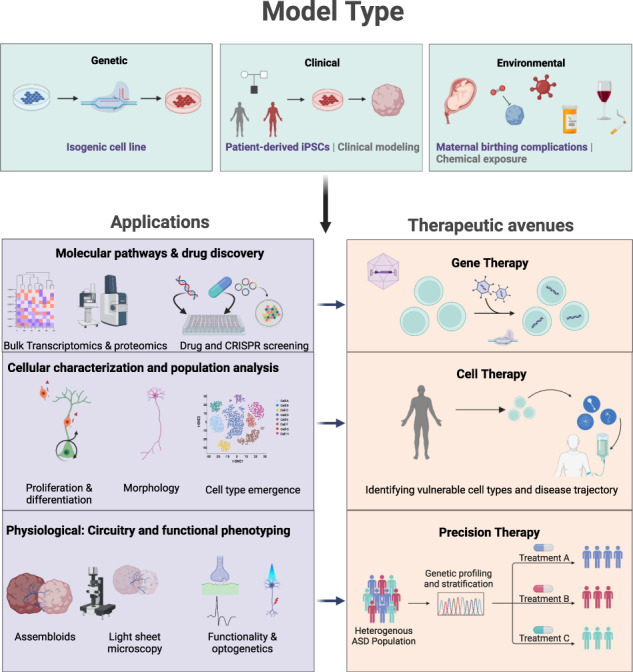
Applications of hPSC modeling in ASD populations: from the benchtop to the clinic. Schematic representation of human pluripotent stem cell (hPSC) modeling of autism spectrum disorder (ASD). The top panel describes modeling avenues as genetic, clinical, or environmental, which can be assessed on the molecular, biological, and functional level (bottom left panel). Therapeutic outcomes from each of these assessments are described in the bottom right panel.

Migration defects can result in a failure for cellular integration and may contribute to downstream disruptions in cell signaling and activity that is present in ASD models. Disruptions in cell circuitry can be probed on a functional level using tools such as single-cell patch electrophysiology, multielectrode array (MEA) recordings of large neuronal populations, and [two-photon] calcium imaging to record abnormalities in firing patterns. These techniques have been used in the aforementioned migration studies as a means of complimenting the findings, as well as in epileptic studies [90, 104–106] to profile synaptic activity in a more sophisticated manner to include recordings of neural oscillations in both single organoids and assembloid systems [82, 90, 107, 108]. Using MEA recordings paired with traditional fMRI and calcium imaging, researchers have identified epileptiform changes that are unique to the brains of those with ASD and epilepsy [109–111], and have even used the models to explore unconventional pharmaceutical rescue of these abnormalities [90]. This method has also been used to show dosage-dependent responses of brain organoids to convulsant and antiepileptic compounds in seizure liability drug screens [108], demonstrating the utility of this system as a putative translational medicine tool. It is with these tools that researchers and clinicians can begin to understand circuitry pathogenesis in ASD to better provide targeted therapeutics (Fig. 2). hPSC modeling has truly revolutionized the field of ASD research and has enabled scientists to examine the pathophysiology from the top (phenotyping broad structural and growth components) to the integrated circuitry between different cell types, all the way down to the core mechanistic components to the disorder (Table 2).

Despite the versatility of recording neural activity in 3D models, they are not without caveats. Techniques such as MEA and electrophysiology are difficult to scale up, and often record superficial neuronal populations on the direct surface of the organoid. Furthermore, a clear caveat comes from the assembloid system itself, as it includes the merging of regionalized systems that are created independently (and artificially) rather than together as with a truly developing brain. Despite being able to generate innervation and achieve neuronal migration in a biologically relevant manner, the order in which these processes occur does not represent that of the fetal brain and would require a more sophisticated approach (concurrently guiding merged organoids/co-cultures) to better represent the complex development of neural circuitry in a fetal brain. It is possible that the artificial timing is what hinders production of rare neural cell types and more complex circuitry [112]. There is also considerable variability in the cell types produced that later participate in organoid circuitry; select long-term organoid cultures have been shown to produce cortical progenitor-derived interneurons [82] however this is not the case for all protocols [113]. The absence of these cell types may result from a failure to reach maturation, or a lack of guidance factors provided by neighboring cell types or directed in the neural medium [114]. Current organoid protocols differ in terms of media components, extracellular matrix use, embedding, shaking, and even so far as nomenclature itself. The latter of which has recently been addressed in a joint call for a standardized naming system within the larger organoid community [48], and will likely expand to include more universal protocols in years to come.

## Modeling environmental insults in ASD using brain organoids

Prenatal environmental insults, comprising either acquired (e.g., infection, substance use, heavy metal exposure, etc.) or inherent (e.g., vitamin deficiencies, stress, diabetes, etc.) pregnancy and birth complications, have been increasingly linked to NDDs including ASD [115]. However, evidence and specificity of these associations is mostly observational. Organoid systems offer unrestricted temporal access to early human neurodevelopmental milestones to examine these epidemiological associations in vitro through perturbation studies (summarized in Table 3) [115].

**Table 3:** Overview of known and associated environmental insults to ASD modeled using neural organoids.

Environmental insult(s)/exposure	Model 3D system	References	Phenotyping	Key findings/phenotypes	Summary
Glucocorticoid	Unguided	Cruceanu et al. [139]	IF, bulk/single-cell RNA transcriptomics, RT-qPCR	Acute exposure to glucocorticoids induced global GR expression/activity, but cell-type-specific transcriptional GC response. Treated organoids exhibited a transcriptional enrichment for behavioral and neurodevelopment-associated genes	Model highlights the association between environmental exposure and genetics on early neurodevelopment
Endomorphin; WIN 55,212-2; nicotine; cortisol; IL17a	Guided	Notaras et al. [233]	IF, TMT LC/MS proteomics, hybrid MS metabolomics, flow cytometry	WIN 55,212-2 (cannabinoid) exposure produced signs of neurotoxicity (DNA damage, apoptosis, depletion of newborn and total neurons)	Cannabinoid exposure may interfere with neocortical neurogenesis in developing human forebrain
Acrylamide	Unguided	Bu et al. [234]	IF, bulk RNA transcriptomics, qPCR, WB	Acrylamide exposure resulted in altered transcriptional profile, NRF2-mediated pathway activation, increased cell apoptosis, increased tau hyperphosphorylation, and reduced neuronal differentiation	Transplacental acrylamide exposure may contribute to neurodevelopmental toxicity in fetus
His, Lys, Thr	Unguided	van Berlekom et al. [235]	Brightfield, bulk RNA transcriptomics, WB	Increased Thr, His, and Lys exposure led to reduced organoid size, reduced mTOR activity, and altered gene expression (mTOR, proliferation, immune function)	Early nutritional availability of amino acids may strongly moderate mTOR function in early neurodevelopment
Cadmium	Unguided	Huang et al. [236]	Brightfield, IF. bulk RNA transcriptomics, qPCR, WB, cytokine assay	Cadmium-induced neural apoptosis, activated GFAP^+^ astrocytes, induced IL-6 release, inhibited neural progenitor proliferation, altered gene expression (downregulated cilium-related gene expression, upregulated metallothionein expression), and decreased ciliary length	Heavy metal cadmium induces global and cellular early developmental neurotoxicity
THC	Unguided	Ao et al. [237]	IF, MEA, qPCR, neurite outgrowth assay	THC-exposed organoids exhibited decreased neuronal maturation, decreased CB1 receptor expression, impaired neurite outgrowth, and reduced spontaneous firing	Prenatal THC exposure may result in impaired neurodevelopment
Cocaine	Guided	Lee et al. [132]	IF, RT-PCR	Cocaine-induced CYP3A5-mediated ROS generation, inhibited neocortical progenitor proliferation, promoted premature neuronal differentiation, and impaired neural tissue development	CYP3A5 may act as a therapeutic target in ameliorating prenatal cocaine exposure-induced neurodevelopmental changes
Cytomegalovirus (HCMV)	Unguided	Brown et al. [238]	Brightfield, IF	HCMV infection led to necrosis, atypical architectural organization (lamination), altered maturation, reduced β-tubulin III^+^ neuronal expression, and morphological changes (cysts, large vacuoles)	HCMV-infected neural organoid phenotypes show clear similarities with clinical specimen pathologies
Cytomegalovirus (HCMV)	Unguided	Sun et al. [126]	IF, bulk RNA transcriptomics, RT-qPCR, WB, calcium imaging, MEA	Organoids infected with HCMV exhibited abnormal growth, layering, and calcium imaging. Phenotypes partially rescued by neutralizing antibodies	HCMV infection impairs early neurodevelopment/function that may be mitigated by neutralizing antibody therapeutics
Cytomegalovirus (HCMV)	Unguided	O’Brien et al. [239]	IF, WB, flow cytometry, bulk RNA transcriptomics, RT-qPCR	HCMV-infected organoids expressed downregulated neurodevelopmental pathways regardless of the extent of infection and IE1 status	Infected organoids with HCMV exhibited downregulated neurodevelopmental pathways that may not be best treated by therapeutics solely designed to target viral replication and/or viral protein/gene expression
Cytomegalovirus (HCMV)	Unguided	Sison et al. [240]	IF, RT-PCR, calcium imaging	HCMV exposure disrupted organoid structure (developmental, neural layering markers) and impaired neuronal and astrocytic response to stimuli. Maribavir treatment partially restored structural and functional changes.	HCMV infection may disrupt cortical layering and calcium regulation in early brain development
Diesel particulate matter	Unguided	Bilinovich et al. [135]	Nanopore/ribosomal reduced/single-cell RNA transcriptomics	DPM-exposed organoids demonstrated extensive RNA modifications (altered cytosine methylation in outer radial glial oxidative mitochondrial transcripts), changes in gene expression (altered oxidative phosphorylation in outer radial glia/other cell types)	DPM exposure may perturb typical mitochondrial function and cellular respiration, resulting in neurodevelopmental changes
Di-(2-ethylhexyl) phthalate (DEHP)	Unguided	Yang et al. [241]	Brightfield, IF, bulk RNA transcriptomics, RT-PCR, flow cytometry	Organoids exposed to DEHP exhibited reduced proliferation, disrupted cell migration, altered cell-ECM interactions, and increased apoptosis	Prenatal exposure to DEHP may induce neurodevelopmental toxicity
Ethanol	Unguided	Zhu et al. [136]	IF, bulk RNA transcriptomics, RT-PCR, neurite outgrowth assay	Ethanol exposure led to attenuated neurite outgrowth, impaired cell proliferation and neural maturation (hyper differentiation of glutamatergic neurons), increased cell death, and altered gene expression (GSX2, RSPO2, Hippo signaling)	Ethanol-impaired neurogenesis may be influenced by early disruption of neuronal subtypes and the skewing of excitatory/inhibitory neuronal populations
HIV-1	Guided; Co-culture	Dos Reis et al. [128]	IF, ELISA, cytotoxicity assay, RT-qPCR	HIV-1-infected organoids demonstrated increased inflammatory responses (TNFa, IL-1b), neuronal loss, and increased cytotoxicity	Incorporating microglia into organoid systems shows promise for modeling hallmarks of HIV-1 neuropathology
HSV-1	Unguided	Qiao et al. [242]	IF, RT-PCR	Organoids infected with HSV-1 exhibited impaired neuronal differentiation and structure (layering, regionalization), as well as increased microglial proliferation/activation and upregulated inflammatory cytokines (TNFα, IL-6, IL-10, IL-4)	HSV-1 organoid infection impairs human neural development and supports the neurodevelopmental disorder pathogen hypothesis
HSV-1	Unguided	Qiao et al. [243]	Brightfield, IF, RT-PCR, bulk RNA transcriptomics	HSV-1 organoid infection led to increased amyloid beta deposition, reactive gliosis, and neuroinflammation (phenotypes partially rescued by Ribavirin or Valacyclovir), as well as neuronal loss	HSV-1 introduction led to multiscale neuropathological phenotypes (neural loss, inflammation, etc.)
HSV-1	Guided	D’Aiuto et al. [127]	Brightfield, IF, RNAscope in situ hybridization, qPCR	HSV-1 reactivation induced neuronal morphological changes (neuronal process degeneration, cell-cell fusion, neuronal syncytia)	3D neural organoids offer a promising opportunity to model HSV-1 CNS infection
HSV-1	Guided; Co-culture	Qiao et al. [244]	IF, ELISA, RT-qPCR	Choroid plexus-like organoid epithelial cells were highly vulnerable to HSV-1 infection, but the introduction of microglia partly induced a protective effect (cGAS-STING pathway)	The cGAS-STING pathway may play important antiviral role in vulnerable choroid plexus
Influenza; Enterovirus; Severe Fever with Thrombocytopenia Syndrome Virus (SFTSV)	Unguided	Zhang et al. [245]	IF, bulk RNA transcriptomics, ELISA, RT-PCR, MEA	Infected organoids exhibited altered size (influenza - reduced; enterovirus - increased), targeted MAP2^+^ neurons (WSN), induced apoptosis of NSCs/neurons (WSN), promoted neuroinflammation (WSN), and altered gene expression (WSN). Specific neurotrophic factors and PYC-12 partly showed neuroprotective effects	WSN infection preferentially infected MAP2^+^ neurons and induced apoptosis in NSCs/neurons, but not astrocytes
Japanese Encephalitis Virus (JEV)	Guided	Zhang et al. [246]	IF, path-clamp, ELISA, WB, RT-qPCR	JEV infection promoted reduced cell proliferation, increased cell death, and resultantly smaller organoid size. JEV preferentially targeted astrocytes and neural progenitors (oRGCs), while exhibiting a greater antiviral response at later stages of development	JEV infection may produce more severe outcomes in younger individuals by preferentially targeting oRGCs and avoiding the greater antiviral response that occurs at later developmental stages
Lyme Neuroborreliosis (LNB)	Guided	Adams et al. [247]	IF	LNB-infected blood-brain barrier-like organoids showed swelling (loss of tight junctions) and reduced structural integrity	Human blood-brain barrier-like organoids support LNB infection and offer promising advantage to elucidate cellular and molecular mechanisms of spirochetal CNS invasion
Heme (malaria)	Unguided	Harbuzariu et al. [248]	IF, apoptosis/necrosis assay	Developing organoids exhibited altered ERBB4/NRG-1, CXCL-10/CXCR3, and BDNF expression in (1) neutral stem and mature cells and (2) following heme-induced neuronal injury. Treatment-induced increased cell death and structural changes that were attenuated with NRG-1 treatment.	NRG-1 may induce neuroprotective effects following heme-induced injury/malaria
Methadone	Guided	Yao et al. [249]	Brightfield, IF, MEA, path-clamp	Methadone-treated organoids displayed reduced growth, suppressed neural network activity, and reduced sodium current/synaptic transmission	Methadone impairs neural growth and function during early neurodevelopment
Methadone	Guided	Wu et al. [250]	Brightfield, IF, patch clamp	Organoids exposed to methadone exhibited reduced neuronal passive function, suppressed increase in membrane neuroexcitability, and disrupted sodium/potassium channel current properties	Prolonged methadone exposure may promote delayed fetal brain development/maturation
Nicotine	Unguided	Wang et al. [131]	IF, qPCR, neurite outgrowth assay	Nicotine-exposed organoids showed premature neuronal differentiation, increased TUJ1^+^ neuron expression, impaired regionalization, and disturbed neurite outgrowth	Nicotine exposure during gestation may disrupt early brain neurogenesis
Nitric oxide (NO)	Unguided	Mokry et al. [251]	Brightfield, IF	NO-induced disrupted tissue organization in neural organoids	Despite its antiviral activity against HCMV, NO may promote developmental deficits during early fetal brain development
Opioid	Guided	Cai et al. [252]	IF, qPCR, MEA	Opioid-treated organoids displayed downregulation of u-opioid receptor expression and altered neural activity reminiscent of opioid tolerance and hyperalgesia	Human spinal microphysiological systems may hold value for furthering exploring spinal cord development following prologued opioid exposure
Phenylalanine	Unguided	Kim et al. [253]	Brightfield, IF, bulk RNA transcriptomics, calcium imaging, myelination quantification	Phenylalanine induced a reduction in organoid size, increase in apoptosis, greater depletion of neural progenitor cells, and altered gene expression (upregulated apoptosis/inflammation pathways, downregulated cell cycle, and amino acid metabolism associated genes). At elevated concentrations, treated organoids exhibited reduced cortical rosette thickness and a decrease in intermediate zone myelination	High phenylalanine exposure may contribute to impaired cortical expansion, myelination lesions, and microencephaly during human brain development
*Plasmodium falciparum* HRP2	Unguided	Harbuzariu et al. [254]	IF, WB, gene array	HRP2 induced increased cell death, inflammation, and tissue disorganization in organoids. Upregulated TLR1/TLR2 expression and exogenous NRG-1 treatment mediated HRP2 effects	NRG-1 may be an effective therapeutic target against malaria-associated HRP2-induced brain injury/inflammation
SARS-CoV-2	Guided	Jacob et al. [121]	IF, bulk RNA transcriptomics, viral titering, qPCR	Despite spare infection of neurons and astrocytes, choroid plexus epithelial cells were robustly infected by SARS-CoV-2 (increased cell death, transcriptional dysregulation associated with inflammatory response and impaired cellular function)	Developing choroid plexus epithelial cells may be a vulnerable cell population to SARS-CoV-2 infection
SARS-CoV-2	Guided; Co-culture	Wang et al. [255]	IF, single-cell RNA transcriptomics, quantitative mass spectrometry, RT-qPCR, plaque assay	Pericyte-like cells preferentially served as SARS-CoV-2 “replication hubs” before propagation to astrocytes and mediating increased inflammatory transcriptional responses (type I interferon)	Pericyte-like cells may act as a niche for productive SARS-CoV-2 infection in the developing CNS
SARS-CoV-2	Guided	Andrews et al. [256]	IF, qPCR	SARS-CoV-2 robustly infected astrocytes in gliogenic organoids (week 22 onward)	SARS-CoV-2 may preferentially infect astrocytes during gliogenesis
SARS-CoV-2 pseudovirus	Guided	Yi et al. [257]	IF, WB	SARS-CoV-2 minimally infected neural layers and sustained expression during development, partially in the soma of mature neurons (ACE2^+^ receptor expression) but also in axons (ACE2^-^)	Neural organoids serve as a useful tool for preliminary investigation into the susceptibility and pathology of SARS-CoV-2 CNS infection
SARS-CoV-2	Unguided; Co-culture	Samudyata et al. [258]	IF, qPCR, single-cell RNA transcriptomics	SARS-CoV-2-infected organoids exhibited increased cell death, decreased postsynaptic termini, and an upregulation of genes associated with interferon response, migration, and synapse engulfment	Despite showing limited neurotropism, SARS-CoV-2 infection may induce a disruption in brain circuit integrity through microglia-mediated synapse elimination
SARS-CoV-2	Guided	McMahon et al. [259]	IF, RT-qPCR	Neural organoids were infected at low viral titers within 6 hr, with a preferential niche in glial and choroid plexus cells. At 14 days post-infection, cleaved caspase-3 co-localized with SARS-CoV-2.	SARS-COV-2 may preferentially target glial cells in developing brain
Sevoflurane	Unguided	Lee et al. [260]	Brightfield, IF, RT-qPCR, ELISA	Organoids exhibited a transient decrease in ventricular zone structures and an upregulation of TUJ1^+^ and MAP2^+^ expression mid-development, but this eventually settled to normal phenotypes over time	Maternal sevoflurane exposure during early gestation may induce abnormal neuronal differentiation during fetal brain development, but may not having lasting impacts
Toxoplasma gondii	Unguided	Seo et al. [125]	IF, bulk RNA transcriptomics, live imaging, electron microscopy	*T. gondii*-infected neural organoids displayed an upregulated type I interferon immune response and altered gene expression associated with protozoan invasion and replication	3D neural organoids are a promising, physiologically-relevant model to investigate the effect of *T. gondii* on human neurodevelopment
Valproic acid	Unguided	Zang et al. [261]	Brightfield, IF, bulk RNA transcriptomics, RT-qPCR, WB	VPA exposure caused reduced organoid size, impaired proliferation/expansion of NPCs (i.e., oRGs), and influenced ASD-risk gene expression (some overlap with irregulated genes identified in ASD patient-derived brains/neural organoids)	VPA-induced teratogenic pathways may be effectively modeled with neural organoids to determine their potential contribution to ASD pathogenesis
Valproic acid	Guided	Cui et al. [134]	IF, bulk RNA transcriptomics, RT-qPCR	Organoids treated with VPA demonstrated increased neural progenitors, inhibited neural differentiation, impaired regionalization, and altered gene expression (some overlap with ASD-associated genes from patient-derived brains/organoids)	Neurodevelopmental disruption as exhibited in VPA-treated neural organoids may contribute to postnatal neurological disorders including ASD
ZIKV	Guided	Qian et al. [56]	Brightfield, IF	ZIKV-infected organoids exhibited a preferential ZIKV tropism for NPC, reduced neuronal cell-layer volume resembling microencephaly	ZIKV exposure during pregnancy may impair early neurogenesis
ZIKV	Unguided	Watanabe et al. [262]	IF, bulk RNA transcriptomics, RT-qPCR	ZIKV infection produced widespread progenitor apoptosis, reduced size, and activated innate immune responses. TIM1, TYRO3 and MER were identified as candidate ZIKV receptors in early developing brain.	Neural organoids are promising models for recapitulating ZIKV fetal infection and studying anti-ZIKV therapies
ZIKV	Guided	Xu et al. [263]	Brightfield, IF, RT-qPCR	ZIKV primarily infected SOX2^+^ NPCs in the VZ and SVZ, resulting in VZ layer thinning, increased cell death, and reduced organoid size. Enoxacin treatment (RNAi enhancer) attenuated viral replication and partially restored normal proliferation levels and layering.	RNAi-mediated therapeutics (e.g., enoxacin) may circumvent ZIKV-induced damage in developing neural tissue
ZIKV-encoded NS2A	Guided	Yoon et al. [264]	IF	ZIV-NS2A disrupts RGC proliferation and adherens junction formation	ZIKV infection may impair early human cortical neurogenesis by targeting and impairing RGC proliferation
ZIKV	Unguided	Dang et al. [117]	Brightfield, IF, RT-qPCR, viral titer assay	Neural organoid ZIKV treatment infects NPCs and activates TLR3, which resultantly triggers apoptosis and blocks neurogenesis	ZIKV-mediated TLR3 activation may underly disrupt cell fate and reduce neural volume
ZIKV	Unguided	Janssens et al. [265]	IF, DNA methylation transcriptomics, RT-PCR, flow cytometry	ZIKV infection alters neural progenitor, astrocyte, and differentiated neuron DNA methylation in genes associated with other neurological disorders (intellectual disability, schizophrenia)	ZIKV infection during early brain development may contribute to delayed-onset neuropsychiatric complications
ZIKV; HSV-1	Unguided	Krenn et al. [266]	IF, bulk RNA transcriptomics, ELISA, RT-qPCR	ZIKV and HSV-1-infected organoids exhibited reduced size, impaired morphology, distinct transcriptional signatures, and differential engagement of the interferon system. Distinct type I interferons could rescue infected organoid phenotypes.	Neural organoids demonstrated phenotypes previously undetected in 2D cultures, thereby supporting their use as a beneficial tool to model viral infections
ZIKV	Unguided	Cugola et al. [119]	Brightfield, IF	ZIKV infection results in reduced proliferative zones and disrupted cortical layering	ZIKV transplacental infection may cause microencephaly via targeting cortical progenitor cells and inducing cell death

*GC* glucocorticoid, *IF* immunofluorescence, *TMT LC/MS* tandem mass tag liquid chromatography–mass spectrometry, *(RT)-(q)PCR* (reverse transcription)-(quantitative) polymerase chain reaction, *WB* western blot, *MEA* multielectrode array, *THC* tetrahydrocannabinol, *ROS* reactive oxygen species, *HCMV* human cytomegalovirus, *DPM* diesel particulate matter, *ASD* autism spectrum disorder, *DEHP* bis(2-ethylhexyl) phthalate, *ECM* extracellular matrix, *HIV-1* human immunodeficiency virus-1, *HSV-1* herpes simplex virus-1, *ELISA* enzyme-linked immunosorbent assay, *SFTSV* severe fever with thrombocytopenia syndrome virus, *NSC* neural stem cell, *JEV* Japanese encephalitis virus, *(o)RGC* (outer) radial glial cell, *LNB* lyme neuroborreliosis, *NO* nitric oxide, *ZIKV* Zika virus, *NPC* neural progenitor cell, *RNAi* RNA interference.

Since 2016, 3D hPSC models have been used to study the effect of infectious agents on early brain development. Maternal infection during pregnancy has been associated with an increased risk of ASD in offspring [116], and strong pathogen-host specificity has previously hindered the potential of traditional animal models to reliably recapitulate human transplacental and intrauterine infection [115]. The advantage of brain organoids to model environmental insults first became apparent in 2016, when the cellular basis of Zika virus (ZIKV)-associated microencephaly was investigated in human UNOs [117–119]. ZIKV^BR^-infected organoids exhibited a reduced growth rate and average growth area compared to mock-infected controls [119], which allowed investigators to provide supporting evidence of a causal link between the 2015 Brazilian ZIKV outbreak and increased incidences of congenital brain malformations in the surrounding population. More recently, organoid studies have provided critical insight into the virulence and putative cellular tropisms of SARS-CoV-2 infection in the developing brain [120–123], as well as potential therapeutic strategies [124]. Other groups have used UNOs or GNOs to explore the consequences of ToRCH infections (e.g., toxoplasmosis [125], cytomegalovirus [126], herpes simplex virus [127], human immunodeficiency virus [128]) on early neurodevelopment [122].

Growing epidemiological evidence implicates substance use and in utero chemical exposure with an increased risk for ASD [129]. To date, various groups have modeled the developmental effect of early exposure to chemical substances using brain organoids, including alcohol [130], nicotine [131], cocaine [132], heavy metals [133], valproic acid [134], and diesel particulate matter [135]. For instance, UNOs continuously exposed to ethanol from day 10–30 of differentiation exhibited increased apoptosis, impaired neurogenesis, and attenuated neurite outgrowth [136]. Furthermore, 2-month UNOs exposed to alcohol levels comparable to binge drinking displayed apoptosis in a cell-type-specific manner, increased metabolic stress, and altered gene expression in key pathways implicated in various neurological diseases [130]. Scalable organoid-based toxicological screens have also shown promise in identifying and assessing the cellular basis of species-specific neurotoxicity [137].

Parental factors and pregnancy complications have also been associated with ASD diagnosis [138]. Organoids allow researchers to investigate the influence of discrete environmental stressors in a controlled environment and precise genetic background, such as maternal stress (e.g., induced by glucocorticoid hormones [139]) and birth complications (e.g., hypoxia [140]). However, despite their clear advantages, organoid modeling of environmental programming is constrained by their inherent limitations. Groups should thoroughly consider the biological implications of missing cell diversity and circuitry during project design and interpretation. For example, 3D cellular models lack intrinsic maternal protective barriers (e.g., placenta, blood-brain separation, xenobiotic inactivation, etc.) that play an essential role in preserving neurodevelopment against environmental insults and may themselves be influenced during environmental programming [115]. Likewise, current environmental perturbation studies using a direct application of a given insult (e.g., toxin) to organoids is not physiologically representative and likely causes exacerbated effects. However, future studies could improve this using organoid transplanted into rodent models where physiological concentrations and drug metabolic processing may be better obtained.

## Profiling developmental trajectories across time

Two of the major barriers to ASD therapeutics are a lack of available biomarkers and a poor understanding of disease trajectory. Prior to the use of human-derived hPSC modeling, scientists were limited to postmortem brain tissue to identify neural biomarkers for ASD, which are subject to degradation and often depict less relevant developmental timepoints. Less invasive imaging techniques can be used at more pertinent timepoints, and even across a developmental continuum, however their low resolution fails to capture relevant biological pathways and the repeated measures across time are often between different individuals and underpowered to provide a conclusive understanding of the brain in a disease context. This has been addressed in recent longitudinal imaging studies [141, 142], but require more patient representation to capture the spectrum of the disorder. Capturing the disease trajectory is further complicated by the time at which ASD diagnoses occur; behavioral abnormalities are likely present well after the disorder and many important neurodevelopmental processes are established [2]. For example, deficits in neuron migration or differentiation may be identified at a later time point, but the causative cell population or biological pathways will remain unidentified using basic clinical assessments. Similarly, many critical synaptic pruning events that are disrupted in ASD and identified through MRI are undetectable by adulthood [143], indicating a critical developmental window that must be more thoroughly examined. The identification of vulnerable windows in development can better guide when populations should be assessed for biomarkers. Should biomarkers be identified during a pre-symptomatic period, at-risk children could be better supported, which is critical when considering the increased risk factor for neuropsychiatric illnesses for individuals with ASD diagnosis later in adulthood [144]. 3D organoids faithfully produce cell types in a spatiotemporal manner similar to that of a developing brain, and so disruptions in any of these processes can be assessed spatially, functionally, and through multi-omic approaches (Fig. 2).

Bulk RNA sequencing can be used to elucidate affected pathways in both pre and postnatal development to identify mechanisms underlying alterations in developmental trajectories [111, 145, 146]. In 1- to 3-month-old cortical GNOs, bulk RNA transcriptomic signatures demonstrated that 1-month-old organoids most closely resembled early mid-fetal (13–16 gestational weeks) through late mid-fetal (19–2 gestational weeks) periods, whereas 3-month-old organoids capture profiles of late mid-fetal (19–24 gestational weeks) through neonatal-early infancy (up to 6 months postnatal) developmental periods. The ability of this model to mirror developmental windows across time lends itself to studying early developmental processes, particularly prenatal neurodevelopment that were previously inaccessible using human samples. Downstream patient clinical information can then be combined with these models to guide core developmental questions surrounding neurodevelopment.

Bulk RNA sequencing was also used in patient-derived forebrain assembloids from individuals with Timothy Syndrome, a monogenic form of ASD. Using ventral tissue at multiple timepoints, the team identified alterations in GABAergic signaling at early differentiation stages where they had previously identified disruptions in interneuron migration [57]. They then used weighted gene co-expression network analysis (WGCNA) after gene set enrichment analysis to generate modules of highly correlated genes within the datasets. From these modules, they concluded that GABAergic signaling disruption was likely due to dysregulation of calcium signaling, which was rescued pharmacologically. Taken together, this transcriptomics approach identified early windows in development that are impaired in models of TS syndrome and further distilled the disruptions down to core mechanistic pathways amendable to pharmacological rescue.

Similarly, RNA sequencing in patient-derived brain organoids was used in the common 22q11.2 CNV to profile developmental processes across 100 days of development. This CNV presents highly variable clinical presentations, ranging from cardiac impairments to general developmental delays [147]. Multiple timepoints were used to capture disruptions in biologically relevant pathways such as pattern specification, NPC proliferation, membrane potential regulation, and glial differentiation [148]. The authors were able to identify biologically relevant windows sensitive to neuronal excitability, which they corroborated with functional assays such as single-cell electrophysiology and calcium imaging. Despite the high variability in clinical presentations, these cellular phenotypes remained consistent across multiple patient-derived lines and were even recapitulated with the heterozygous KO of a single gene, *DGCR8*. This demonstrates the versatility of using organoids to explore potential therapeutic avenues and driver genes within CNVs.

Bulk transcriptomics can identify unbiased biological pathways and biomarkers that may otherwise be missed with conventional phenotyping methods such as immunofluorescence or single gene expression. One notable disadvantage to the technique is the homogenization of highly heterogeneous tissue to capture the average global gene expression; in doing so, subtle intracellular signatures among heterogeneous populations are overlooked. Single-cell RNA sequencing (scRNA Seq) is an alternative transcriptomic approach to capture cell-type identity and individual transcriptomic profiles and trajectories over time. Downstream analyses have been aided with the release of publicly available databases, which include hPSCs, ESCs, embryoid bodies, and neural organoids at multiple developmental timepoints [149, 150]. Information from these databases can be combined with spatial anatomical tools such as the Allen Brain Atlas to provide reliable cell-type identification and pseudotemporal gene expression alignment for identification of developmental trajectories. More recently, scRNA Seq has been combined with lineage tracing inducible CRISPR technology, termed iTracer, to identify cell lineage dynamics and clonality across UNO development [151]. This technique introduces a barcoding library to identify cell types from an hPSC pool, which is retained in daughter cells throughout division and differentiation, and when paired with an inducible CRISPR scar can track lineage dynamics during a discrete window of time. This can putatively be used to identify small windows of changes to cellular fate during UNO development and can be complemented with techniques such as 4D light sheet microscopy to track migration of daughter cells and newly generated neurons. This platform identified lineage disruptions in a neurodevelopmental dysplasia KO model [151], which was paired with 4D spatial sequencing to show disruptions in brain regionalization consistent with these lineage disruptions. Using sophisticated lineage tracing in a heterogeneous human model enables us to ask questions about population-specific dynamics throughout space and time—formerly something that was restricted to animal models.

Understanding transcriptomics on a single-cell level compliments the diversity of cell types that organoids can produce and has helped establish vulnerable cell-type populations to ASD [152, 153]. The exact localization of these cell types in an ASD model has yet to be fully established but can be aided with the use of spatial sequencing to determine the cytoarchitectural microenvironment within individuals with ASD. A recent study examined the adult human cortex using 10x Genomics’ barcoding platform, Visium, to generate spatial maps of gene expression within the human dorsolateral prefrontal cortex. When this dataset was integrated with other NDD datasets, including those of ASD patients, there was a profound layer-specific enrichment of known ASD genes, highlighting the need to understand ASD genetics both spatially and functionally [154]. Defining cellular transcriptomes with spatial resolution is especially relevant when using 3D models that establish brain laminar structure and distinct cytoarchitecture. To this end, spatial transcriptomics lends an unbiased perspective on cell-type-specific abnormalities through generation of spatial gene maps that, when paired with imaging techniques such as MRI, could help delineate structural abnormalities and underlying circuit defects not identified through scRNA Seq alone. Cell population microenvironments can even be probed for activity-dependent pathways to help establish the affected circuits and their regionalization. Spatial transcriptomics have been used in organoids to establish neural lineage dynamics with spatial resolution (iTracer), neurodevelopmental patterning factors [155], and can be paired with fluorescent tagging to isolate or identify specific regionalization within heterogeneous organoid or diseased tissue [156].

The use of transcriptomic profiling provides powerful information about cell identity, lineage, and localization. Analysis pipelines enable the user to infer cell trajectory, intercellular communication [157], and can even be used to predict drug response [158, 159]. Recent developments in electrophysiology can also allow a glimpse into the synaptic activity of a given cell via Patch-Seq, a modified version of whole-cell patch-clamp electrophysiology that enables transcriptomic capturing as well as morphological rendering of a given cell. This three-in-one platform provides comprehensive information about the functionality of a cell as well as its operative biological pathways [160] and could have powerful implications in understanding ASD pathophysiology. It is a low-throughput alternative, however, and should be used selectivity within cell populations that are known to be disrupted in the disorder.

Another tool to capture functionality across time includes multielectrode arrays, which are capable of recording neural populations in 2D and intact 3D cultures across development. Importantly, these recordings are done in an unbiased manner to capture population-wide recordings and synchronous activity of diverse neural populations [107, 108]. MEA recordings in cortical organoids have been shown to correlate with that of human preterm neonatal EEG signatures [82], and can therefore provide a glimpse into the network activity of ASD populations during critical developmental windows. Of note, these oscillations can even be captured in assembloid systems [90], offering the ability to capture inter-organoid circuitry, generalized EEG patterns between both organoids, as well as focal signatures to a particular brain region [after stimulating the other]. Knowledge of how particular brain regions are affected functionally can help clinicians decide which pharmaceutical approaches may be most beneficial to their patients [161–163]. They can provide screening opportunities for clinicians to modify pharmaceutical compounds in a controlled environment to target ASD-specific pathways [82, 164–166], and can even identify causative driver genes that can be targeted by AAV- or ASO- based gene therapy [166–173]. Following refinement and rigorous testing, patient-derived neural organoids can be used to enhance and personalize cell therapies, gene therapy, and drug discovery, thereby accelerating their transition from the benchtop to the clinic (Fig. 2).

## Limitations to human modeling and future directions

2D and 3D human models have made enormous progress in the past decade; with the emergence of stem cell reprogramming, patient-derived skin and blood samples are now capable of producing hPSCs that can later go on to mimic general and brain-region-specific processes. These models have great potential for clinical applications and to understand the mechanisms of ASD pathology. They are not without their caveats, however, which include limitations to growth, tissue maturity, and an absence of vascularization and external input from the peripheral nervous system (summarized in Table 1).

Brain organoids have undergone extensive transcriptomic profiling to show the presence of many different brain-region cell types that emerge in a spatial-temporal manner [44]. Multiple studies have revealed the persistence of a stem cell niche alongside these mature cell types, which supports the use of brain organoids to model fetal development [111]. The presence of this niche is unique to organoids and makes late-stage developmental modeling difficult to achieve. In addition, long-term cultures are further hindered by a lack of vascularization and nutrient flow to the inner organoid core [174], which is compounded by the absence of the blood-brain barrier and its inclusion of immune cells such as microglia. This is especially a limitation to modeling autism, as microglia are a proposed vulnerable cell type within ASD and are thought to contribute towards its immunopathology [175, 176]. Luckily this caveat has been addressed with the introduction of blood vessel organoids that provide vascularization networks at the cellular level, which in turn increase NPC populations, and introduce microglia into the environment [177, 178]. This is incredibly important given the prenatal time point where neurovascularization occurs, the human-specific expression pattern of vascular cells, and its influence on brain structure and development [179].

Microglia populations have also been incorporated into growing organoids through direct co-culture or merging of NPCs and primitive macrophages, which are capable of synaptic pruning and phagocytic activity once mature. These models can be used to investigate the effects of the immune environment on brain pathology [180, 181]. The addition of microglia would provide critical developmental cues to all cell types in the organoids, while supplying a cellular substrate to understand how neuroinflammatory processes occur in NDDs. For example, over-pruning of synapses is one type of deleterious function of abnormal microglia that have yet to be modeled in organoids and would allow complex modeling in 3D.

Despite the enormous progress in modeling specific brain regions through guided differentiation, an element of the CNS that has been underrepresented in human ASD research is the eye. Multiple NDDs are associated with vision disorders, and there has been tremendous advancement of retinal organoid protocols. Retina morphogenesis is a highly regulated process both temporally and spatially, and much like the developing brain requires a stem cell niche that is present in early development [182–184]. Individuals who are blind are at least ten times as likely to have ASD, and clinical studies have shown comorbid vision impairment within ASD populations, although the underlying pathogenesis between these two conditions remains unexplained [185, 186]. Retinal organoids are capable of producing retinal pigment epithelia and functional photoreceptors, and their application to ASD modeling would provide novel insights into how retinal development may be impaired and later give rise to visual impairment and dysfunction. Further, the emergence of retinal-cortical assembloids [187], provides the necessary tool to study eye-brain connections in NDDs to understand how dysfunctional sensory input and function may arise.

In recent years, growing evidence suggests the importance of exploring ASD models beyond the CNS due to the high proportion of sensory dysfunctions reported amongst individuals with ASD (as high as 90% [188]). Mouse and fly studies highlight the role of the somatosensory nervous system in ASD sensory and behavioral deficits [189–192]. Building from the knowledge gained by 2D cultures, dorsal root ganglion-like organoids [193] and neuromuscular organoids [194, 195] have emerged and offer a promising opportunity to investigate the role of the PNS in ASD pathophysiology, such as altered sensory functioning. This approach would integrate external input into what has traditionally been a CNS-exclusive model, providing a more complete understanding of ASD pathogenesis.

An exciting model to examine developmental biology more comprehensively, and with theoretical inclusion of all the systems noted above, include synthetic embryos, or embryoids. These novel systems produce gastrulating embryo-like structures that are capable of undergoing organogenesis [196, 197]. While prototypes have been formed in mouse ESCs, and can only reach 8 days of development, optimizations in a human background may achieve month-long growth periods that would enable scientists a novel glimpse into human fetal development (reviewed in ref. [198]). An important consideration, however, would be the inclusion of stimuli to mimic true gastrulation both within and external to the womb. Notwithstanding, these exciting advancements also give rise to several important ethical considerations. While there have been some preliminary discussions concerning hPSC-based ethics in research [199–202], these discussions must be formalized, informed by science, and made jointly between experts in the field, policymakers, and activists in order to develop appropriate universal standards.

While each of the models discussed in this review provide novel insight into neural development and circuitry, they remain undoubtedly limited by the natural heterogeneity within both the model itself and the clinical pathophysiology of ASD. It is more likely that these models will provide a starting point for understanding ASD pathogenesis that, when coupled with a multitude of animal and clinical modeling, may ultimately result in a therapeutic breakthrough [203]. Importantly, we also acknowledge the essential role that members of the ASD community play in conducting thoughtful and meaningful research. Self-advocates have expressed their need for improved social support systems, and we hope that the incorporation of those personally affected into decision making will bolster the research done at the bench, and ultimately provide a more comprehensive and compassionate approach to addressing ASD therapeutics and clinical outcomes [204–208]. Given the accelerated pace of brain organoid research over the last few years, this human and patient-specific model system will undoubtedly play a critical role in helping to develop future therapies.

## References

[1] Maenner MJ, Shaw KA, Bakian AV, Bilder DA, Durkin MS, Esler A (2021). Prevalence and characteristics of autism spectrum disorder among children aged 8 years—autism and developmental disabilities monitoring network, 11 sites, United States, 2018. MMWR Surveill Summ.

[2] Association AP. Diagnostic and statistical manual of mental disorders: DSM-5, 5th edn. American Psychiatry Association; Arlington, 2013.

[3] Mazarati AM, Lewis ML, Pittman QJ (2017). Neurobehavioral comorbidities of epilepsy: role of inflammation. Epilepsia.

[4] Bozzi Y, Provenzano G, Casarosa S (2018). Neurobiological bases of autism-epilepsy comorbidity: a focus on excitation/inhibition imbalance. Eur J Neurosci.

[5] Zeidan J, Fombonne E, Scorah J, Ibrahim A, Durkin MS, Saxena S (2022). Global prevalence of autism: a systematic review update. Autism Res.

[6] McElhanon BO, McCracken C, Karpen S, Sharp WG (2014). Gastrointestinal symptoms in autism spectrum disorder: a meta-analysis. Pediatrics.

[7] Holingue C, Newill C, Lee LC, Pasricha PJ, Daniele Fallin M (2018). Gastrointestinal symptoms in autism spectrum disorder: A review of the literature on ascertainment and prevalence. Autism Res.

[8] Mutluer T, Aslan Genc H, Ozcan Morey A, Yapici Eser H, Ertinmaz B, Can M (2022). Population-based psychiatric comorbidity in children and adolescents with autism spectrum disorder: a meta-analysis. Front Psychiatry.

[9] Joshi G, Faraone SV, Wozniak J, Tarko L, Fried R, Galdo M (2017). Symptom profile of ADHD in youth with high-functioning autism spectrum disorder: a comparative study in psychiatrically referred populations. J Atten Disord.

[10] Eyring KW, Geschwind DH (2021). Three decades of ASD genetics: building a foundation for neurobiological understanding and treatment. Hum Mol Genet.

[11] Tick B, Bolton P, Happe F, Rutter M, Rijsdijk F (2016). Heritability of autism spectrum disorders: a meta-analysis of twin studies. J Child Psychol Psychiatry.

[12] Uddin M, Unda BK, Kwan V, Holzapfel NT, White SH, Chalil L (2018). OTUD7A regulates neurodevelopmental phenotypes in the 15q13.3 microdeletion syndrome. Am J Hum Genet.

[13] Richter M, Murtaza N, Scharrenberg R, White SH, Johanns O, Walker S (2019). Altered TAOK2 activity causes autism-related neurodevelopmental and cognitive abnormalities through RhoA signaling. Mol Psychiatry.

[14] Satterstrom FK, Kosmicki JA, Wang J, Breen MS, De Rubeis S, An JY (2020). Large-scale exome sequencing study implicates both developmental and functional changes in the neurobiology of autism. Cell.

[15] Scharrenberg R, Richter M, Johanns O, Meka DP, Rucker T, Murtaza N, et al. TAOK2 rescues autism-linked developmental deficits in a 16p11.2 microdeletion mouse model. Mol Psychiatry. 2022; 10.1038/s41380-022-01785-3. 10.1038/s41380-022-01785-3 PMC9734055 36123424

[16] Yan QJ, Asafo-Adjei PK, Arnold HM, Brown RE, Bauchwitz RP (2004). A phenotypic and molecular characterization of the fmr1-tm1Cgr fragile X mouse. Genes Brain Behav.

[17] Zhou X, Feliciano P, Shu C, Wang T, Astrovskaya I, Hall JB (2022). Integrating de novo and inherited variants in 42,607 autism cases identifies mutations in new moderate-risk genes. Nat Genet.

[18] Unda BK, Chalil L, Yoon S, Kilpatrick S, Irwin C, Xing S, et al. Impaired OTUD7A-dependent Ankyrin regulation mediates neuronal dysfunction in mouse and human models of the 15q13.3 microdeletion syndrome. Mol Psychiatry. 2023. 10.1038/s41380-022-01937-5. 10.1038/s41380-022-01937-5 PMC10208958 36604605

[19] Chailangkarn T, Trujillo CA, Freitas BC, Hrvoj-Mihic B, Herai RH, Yu DX (2016). A human neurodevelopmental model for Williams syndrome. Nature.

[20] Deneault E, White SH, Rodrigues DC, Ross PJ, Faheem M, Zaslavsky K (2018). Complete disruption of autism-susceptibility genes by gene editing predominantly reduces functional connectivity of isogenic human neurons. Stem Cell Rep.

[21] Roberts JE, Bradshaw J, Will E, Hogan AL, McQuillin S, Hills K (2020). Emergence and rate of autism in fragile X syndrome across the first years of life. Dev Psychopathol.

[22] Bakker CE, Verheij C, Willemsen R, van der Helm R, Oerlemans F, The Dutch-Belgian Fragile X Corsorthium (1994). Fmr1 knockout mice: a model to study fragile X mental retardation. Cell.

[23] Marshall JJ, Mason JO (2019). Mouse vs man: organoid models of brain development & disease. Brain Res.

[24] Nestler EJ, Hyman SE (2010). Animal models of neuropsychiatric disorders. Nat Neurosci.

[25] Takahashi K, Yamanaka S (2006). Induction of pluripotent stem cells from mouse embryonic and adult fibroblast cultures by defined factors. Cell.

[26] de la Torre-Ubieta L, Won H, Stein JL, Geschwind DH (2016). Advancing the understanding of autism disease mechanisms through genetics. Nat Med.

[27] Zhang W, Ross PJ, Ellis J, Salter MW (2022). Targeting NMDA receptors in neuropsychiatric disorders by drug screening on human neurons derived from pluripotent stem cells. Transl Psychiatry.

[28] Dixon TA, Muotri AR. Advancing preclinical models of psychiatric disorders with human brain organoid cultures. Mol Psychiatry. 2022; 10.1038/s41380-022-01708-2. 10.1038/s41380-022-01708-2 PMC9812789 35948659

[29] Yoon KJ, Nguyen HN, Ursini G, Zhang F, Kim NS, Wen Z (2014). Modeling a genetic risk for schizophrenia in iPSCs and mice reveals neural stem cell deficits associated with adherens junctions and polarity. Cell Stem Cell.

[30] Topol A, English JA, Flaherty E, Rajarajan P, Hartley BJ, Gupta S (2015). Increased abundance of translation machinery in stem cell-derived neural progenitor cells from four schizophrenia patients. Transl Psychiatry.

[31] Sans N, Ezan J, Moreau MM, Montcouquiol M. In: Sala C, Verpelli C, editors. Neuronal and synaptic dysfunction in autism spectrum disorder and intellectual disability. Ch. 13. Academic Press; p. 189–219 2016.

[32] de Anda FC, Rosario AL, Durak O, Tran T, Graff J, Meletis K (2012). Autism spectrum disorder susceptibility gene TAOK2 affects basal dendrite formation in the neocortex. Nat Neurosci.

[33] Courchesne E, Carper R, Akshoomoff N (2003). Evidence of brain overgrowth in the first year of life in autism. J Am Med Assoc.

[34] Dementieva YA, Vance DD, Donnelly SL, Elston LA, Wolpert CM, Ravan SA (2005). Accelerated head growth in early development of individuals with autism. Pediatr Neurol.

[35] Wang M, Wei PC, Lim CK, Gallina IS, Marshall S, Marchetto MC (2020). Increased neural progenitor proliferation in a hiPSC model of autism induces replication stress-associated genome instability. Cell Stem Cell.

[36] Marchetto MC, Belinson H, Tian Y, Freitas BC, Fu C, Vadodaria K (2017). Altered proliferation and networks in neural cells derived from idiopathic autistic individuals. Mol Psychiatry.

[37] Connacher R, Williams M, Prem S, Yeung PL, Matteson P, Mehta M (2022). Autism NPCs from both idiopathic and CNV 16p11.2 deletion patients exhibit dysregulation of proliferation and mitogenic responses. Stem Cell Rep.

[38] Readhead B, Hartley BJ, Eastwood BJ, Collier DA, Evans D, Farias R (2018). Expression-based drug screening of neural progenitor cells from individuals with schizophrenia. Nat Commun.

[39] Caiazzo M, Dell’Anno MT, Dvoretskova E, Lazarevic D, Taverna S, Leo D (2011). Direct generation of functional dopaminergic neurons from mouse and human fibroblasts. Nature.

[40] Chanoumidou K, Hernandez-Rodriguez B, Windener F, Thomas C, Stehling M, Mozafari S (2021). One-step reprogramming of human fibroblasts into oligodendrocyte-like cells by SOX10, OLIG2, and NKX6.2. Stem Cell Rep.

[41] Pang ZP, Yang N, Vierbuchen T, Ostermeier A, Fuentes DR, Yang TQ (2011). Induction of human neuronal cells by defined transcription factors. Nature.

[42] Zhang Y, Pak C, Han Y, Ahlenius H, Zhang Z, Chanda S (2013). Rapid single-step induction of functional neurons from human pluripotent stem cells. Neuron.

[43] Mertens J, Marchetto MC, Bardy C, Gage FH (2016). Evaluating cell reprogramming, differentiation and conversion technologies in neuroscience. Nat Rev Neurosci.

[44] Amin ND, Pasca SP (2018). Building models of brain disorders with three-dimensional organoids. Neuron.

[45] Kelley KW, Pasca SP (2022). Human brain organogenesis: toward a cellular understanding of development and disease. Cell.

[46] Qian X, Song H, Ming GL. Brain organoids: advances, applications and challenges. Development. 2019;146:dev166074. 10.1242/dev.166074 PMC6503989 30992274

[47] Lancaster MA, Knoblich JA (2014). Generation of cerebral organoids from human pluripotent stem cells. Nat Protoc.

[48] Pasca SP, Arlotta P, Bateup HS, Camp JG, Cappello S, Gage FH (2022). A nomenclature consensus for nervous system organoids and assembloids. Nature.

[49] Lancaster MA, Renner M, Martin CA, Wenzel D, Bicknell LS, Hurles ME (2013). Cerebral organoids model human brain development and microcephaly. Nature.

[50] Camp JG, Badsha F, Florio M, Kanton S, Gerber T, Wilsch-Brauninger M (2015). Human cerebral organoids recapitulate gene expression programs of fetal neocortex development. Proc Natl Acad Sci USA.

[51] Luo C, Lancaster MA, Castanon R, Nery JR, Knoblich JA, Ecker JR (2016). Cerebral organoids recapitulate epigenomic signatures of the human fetal brain. Cell Rep.

[52] Pasca AM, Sloan SA, Clarke LE, Tian Y, Makinson CD, Huber N (2015). Functional cortical neurons and astrocytes from human pluripotent stem cells in 3D culture. Nat Methods.

[53] Lancaster MA, Corsini NS, Wolfinger S, Gustafson EH, Phillips AW, Burkard TR (2017). Guided self-organization and cortical plate formation in human brain organoids. Nat Biotechnol.

[54] Xiang Y, Tanaka Y, Patterson B, Kang YJ, Govindaiah G, Roselaar N (2017). Fusion of regionally specified hPSC-derived organoids models human brain development and interneuron migration. Cell Stem Cell.

[55] Eiraku M, Watanabe K, Matsuo-Takasaki M, Kawada M, Yonemura S, Matsumura M (2008). Self-organized formation of polarized cortical tissues from ESCs and its active manipulation by extrinsic signals. Cell Stem Cell.

[56] Qian X, Nguyen HN, Song MM, Hadiono C, Ogden SC, Hammack C (2016). Brain-region-specific organoids using mini-bioreactors for modeling ZIKV exposure. Cell.

[57] Birey F, Andersen J, Makinson CD, Islam S, Wei W, Huber N (2017). Assembly of functionally integrated human forebrain spheroids. Nature.

[58] Bagley JA, Reumann D, Bian S, Levi-Strauss J, Knoblich JA (2017). Fused cerebral organoids model interactions between brain regions. Nat Methods.

[59] Mariani J, Simonini MV, Palejev D, Tomasini L, Coppola G, Szekely AM (2012). Modeling human cortical development in vitro using induced pluripotent stem cells. Proc Natl Acad Sci USA.

[60] Jo J, Xiao Y, Sun AX, Cukuroglu E, Tran HD, Goke J (2016). Midbrain-like organoids from human pluripotent stem cells contain functional dopaminergic and neuromelanin-producing neurons. Cell Stem Cell.

[61] Xiang Y, Tanaka Y, Cakir B, Patterson B, Kim KY, Sun P (2019). hESC-derived thalamic organoids form reciprocal projections when fused with cortical organoids. Cell Stem Cell.

[62] Miura Y, Li MY, Birey F, Ikeda K, Revah O, Thete MV (2020). Generation of human striatal organoids and cortico-striatal assembloids from human pluripotent stem cells. Nat Biotechnol.

[63] Ozone C, Suga H, Eiraku M, Kadoshima T, Yonemura S, Takata N (2016). Functional anterior pituitary generated in self-organizing culture of human embryonic stem cells. Nat Commun.

[64] Huang WK, Wong SZH, Pather SR, Nguyen PTT, Zhang F, Zhang DY (2021). Generation of hypothalamic arcuate organoids from human induced pluripotent stem cells. Cell Stem Cell.

[65] Sakaguchi H, Kadoshima T, Soen M, Narii N, Ishida Y, Ohgushi M (2015). Generation of functional hippocampal neurons from self-organizing human embryonic stem cell-derived dorsomedial telencephalic tissue. Nat Commun.

[66] Pellegrini L, Bonfio C, Chadwick J, Begum F, Skehel M, Lancaster MA. Human CNS barrier-forming organoids with cerebrospinal fluid production. Science. 2020;369:eaaz5626. 10.1126/science.aaz5626 PMC7116154 32527923

[67] Muguruma K, Nishiyama A, Kawakami H, Hashimoto K, Sasai Y (2015). Self-organization of polarized cerebellar tissue in 3D culture of human pluripotent stem cells. Cell Rep.

[68] Eura N, Matsui TK, Luginbuhl J, Matsubayashi M, Nanaura H, Shiota T (2020). Brainstem organoids from human pluripotent stem cells. Front Neurosci.

[69] Ogura T, Sakaguchi H, Miyamoto S, Takahashi J. Three-dimensional induction of dorsal, intermediate and ventral spinal cord tissues from human pluripotent stem cells. Development. 2018;145:dev162214. 10.1242/dev.162214 PMC6124545 30061169

[70] Andersen J, Revah O, Miura Y, Thom N, Amin ND, Kelley KW (2020). Generation of functional human 3D cortico-motor assembloids. Cell.

[71] Sparks BF, Friedman SD, Shaw DW, Aylward EH, Echelard D, Artru AA (2002). Brain structural abnormalities in young children with autism spectrum disorder. Neurology.

[72] Stanfield AC, McIntosh AM, Spencer MD, Philip R, Gaur S, Lawrie SM (2008). Towards a neuroanatomy of autism: a systematic review and meta-analysis of structural magnetic resonance imaging studies. Eur Psychiatry.

[73] Richards R, Greimel E, Kliemann D, Koerte IK, Schulte-Korne G, Reuter M (2020). Increased hippocampal shape asymmetry and volumetric ventricular asymmetry in autism spectrum disorder. Neuroimage Clin.

[74] Turner AH, Greenspan KS, van Erp TGM (2016). Pallidum and lateral ventricle volume enlargement in autism spectrum disorder. Psychiatry Res Neuroimaging.

[75] Pan YH, Wu N, Yuan XB (2019). Toward a better understanding of neuronal migration deficits in autism spectrum disorders. Front Cell Dev Biol.

[76] D’Mello AM, Stoodley CJ (2015). Cerebro-cerebellar circuits in autism spectrum disorder. Front Neurosci.

[77] Di Martino A, Kelly C, Grzadzinski R, Zuo XN, Mennes M, Mairena MA (2011). Aberrant striatal functional connectivity in children with autism. Biol Psychiatry.

[78] Hull JV, Dokovna LB, Jacokes ZJ, Torgerson CM, Irimia A, Van Horn JD (2016). Resting-state functional connectivity in autism spectrum disorders: a review. Front Psychiatry.

[79] Li Y, Muffat J, Omer A, Bosch I, Lancaster MA, Sur M (2017). Induction of expansion and folding in human cerebral organoids. Cell Stem Cell.

[80] Dhaliwal N, Choi WWY, Muffat J, Li Y (2021). Modeling PTEN overexpression-induced microcephaly in human brain organoids. Mol Brain.

[81] Birey F, Li MY, Gordon A, Thete MV, Valencia AM, Revah O (2022). Dissecting the molecular basis of human interneuron migration in forebrain assembloids from Timothy syndrome. Cell Stem Cell.

[82] Trujillo CA, Gao R, Negraes PD, Gu J, Buchanan J, Preissl S (2019). Complex oscillatory waves emerging from cortical organoids model early human brain network development. Cell Stem Cell.

[83] Rubenstein JL, Merzenich MM (2003). Model of autism: increased ratio of excitation/inhibition in key neural systems. Genes Brain Behav.

[84] Tuchman R, Rapin I (2002). Epilepsy in autism. Lancet Neurol.

[85] Chen JA, Penagarikano O, Belgard TG, Swarup V, Geschwind DH (2015). The emerging picture of autism spectrum disorder: genetics and pathology. Annu Rev Pathol.

[86] DiCicco-Bloom E, Lord C, Zwaigenbaum L, Courchesne E, Dager SR, Schmitz C (2006). The developmental neurobiology of autism spectrum disorder. J Neurosci.

[87] Penagarikano O, Abrahams BS, Herman EI, Winden KD, Gdalyahu A, Dong H (2011). Absence of CNTNAP2 leads to epilepsy, neuronal migration abnormalities, and core autism-related deficits. Cell.

[88] McVicar KA, Ballaban-Gil K, Rapin I, Moshe SL, Shinnar S (2005). Epileptiform EEG abnormalities in children with language regression. Neurology.

[89] Milovanovic M, Grujicic R (2021). Electroencephalography in assessment of autism spectrum disorders: a review. Front Psychiatry.

[90] Samarasinghe RA, Miranda OA, Buth JE, Mitchell S, Ferando I, Watanabe M (2021). Identification of neural oscillations and epileptiform changes in human brain organoids. Nat Neurosci.

[91] Tierney AL, Gabard-Durnam L, Vogel-Farley V, Tager-Flusberg H, Nelson CA (2012). Developmental trajectories of resting EEG power: an endophenotype of autism spectrum disorder. PLoS ONE.

[92] Bandler RC, Vitali I, Delgado RN, Ho MC, Dvoretskova E, Ibarra Molinas JS (2022). Single-cell delineation of lineage and genetic identity in the mouse brain. Nature.

[93] Delgado RN, Allen DE, Keefe MG, Mancia Leon WR, Ziffra RS, Crouch EE (2022). Individual human cortical progenitors can produce excitatory and inhibitory neurons. Nature.

[94] Bandler RC, Mayer C (2023). Deciphering inhibitory neuron development: the paths to diversity. Curr Opin Neurobiol.

[95] Gelman D, Griveau A, Dehorter N, Teissier A, Varela C, Pla R (2011). A wide diversity of cortical GABAergic interneurons derives from the embryonic preoptic area. J Neurosci.

[96] Wonders CP, Anderson SA (2006). The origin and specification of cortical interneurons. Nat Rev Neurosci.

[97] Rapanelli M, Frick LR, Pittenger C (2017). The role of interneurons in autism and Tourette syndrome. Trends Neurosci.

[98] Fejgin K, Nielsen J, Birknow MR, Bastlund JF, Nielsen V, Lauridsen JB (2014). A mouse model that recapitulates cardinal features of the 15q13.3 microdeletion syndrome including schizophrenia- and epilepsy-related alterations. Biol Psychiatry.

[99] Meechan DW, Maynard TM, Tucker ES, Fernandez A, Karpinski BA, Rothblat LA (2015). Modeling a model: mouse genetics, 22q11.2 Deletion Syndrome, and disorders of cortical circuit development. Prog Neurobiol.

[100] Sanders SJ, He X, Willsey AJ, Ercan-Sencicek AG, Samocha KE, Cicek AE (2015). Insights into autism spectrum disorder genomic architecture and biology from 71 risk loci. Neuron.

[101] Silva AI, Haddon JE, Ahmed Syed Y, Trent S, Lin TE, Patel Y (2019). Cyfip1 haploinsufficient rats show white matter changes, myelin thinning, abnormal oligodendrocytes and behavioural inflexibility. Nat Commun.

[102] Walsh JJ, Christoffel DJ, Heifets BD, Ben-Dor GA, Selimbeyoglu A, Hung LW (2018). 5-HT release in nucleus accumbens rescues social deficits in mouse autism model. Nature.

[103] Bey AL, Wang X, Yan H, Kim N, Passman RL, Yang Y (2018). Brain region-specific disruption of Shank3 in mice reveals a dissociation for cortical and striatal circuits in autism-related behaviors. Transl Psychiatry.

[104] Ammothumkandy A, Ravina K, Wolseley V, Tartt AN, Yu PN, Corona L (2022). Altered adult neurogenesis and gliogenesis in patients with mesial temporal lobe epilepsy. Nat Neurosci.

[105] DeRosa BA, El Hokayem J, Artimovich E, Garcia-Serje C, Phillips AW, Van Booven D (2018). Convergent pathways in idiopathic autism revealed by time course transcriptomic analysis of patient-derived neurons. Sci Rep.

[106] Russo FB, Freitas BC, Pignatari GC, Fernandes IR, Sebat J, Muotri AR (2018). Modeling the interplay between neurons and astrocytes in autism using human induced pluripotent stem cells. Biol Psychiatry.

[107] Foliaki ST, Schwarz B, Groveman BR, Walters RO, Ferreira NC, Orru CD (2021). Neuronal excitatory-to-inhibitory balance is altered in cerebral organoid models of genetic neurological diseases. Mol Brain.

[108] Yokoi R, Shibata M, Odawara A, Ishibashi Y, Nagafuku N, Matsuda N (2021). Analysis of signal components <500 Hz in brain organoids coupled to microelectrode arrays: a reliable test-bed for preclinical seizure liability assessment of drugs and screening of antiepileptic drugs. Biochem Biophys Rep.

[109] Du Y, Fu Z, Xing Y, Lin D, Pearlson G, Kochunov P (2021). Evidence of shared and distinct functional and structural brain signatures in schizophrenia and autism spectrum disorder. Commun Biol.

[110] Griesi-Oliveira K, Acab A, Gupta AR, Sunaga DY, Chailangkarn T, Nicol X (2015). Modeling non-syndromic autism and the impact of TRPC6 disruption in human neurons. Mol Psychiatry.

[111] Urresti J, Zhang P, Moran-Losada P, Yu NK, Negraes PD, Trujillo CA (2021). Cortical organoids model early brain development disrupted by 16p11.2 copy number variants in autism. Mol Psychiatry.

[112] Kanton S, Pasca SP. Human assembloids. Development 2022;149:dev201120. 10.1242/dev.201120 36317797

[113] Gordon A, Yoon SJ, Tran SS, Makinson CD, Park JY, Andersen J (2021). Long-term maturation of human cortical organoids matches key early postnatal transitions. Nat Neurosci.

[114] Mayhew CN, Singhania R (2022). A review of protocols for brain organoids and applications for disease modeling. STAR Protoc.

[115] Sarieva K, Mayer S (2021). The effects of environmental adversities on human neocortical neurogenesis modeled in brain organoids. Front Mol Biosci.

[116] Jiang HY, Xu LL, Shao L, Xia RM, Yu ZH, Ling ZX (2016). Maternal infection during pregnancy and risk of autism spectrum disorders: a systematic review and meta-analysis. Brain Behav Immun.

[117] Dang J, Tiwari SK, Lichinchi G, Qin Y, Patil VS, Eroshkin AM (2016). Zika virus depletes neural progenitors in human cerebral organoids through activation of the innate immune receptor TLR3. Cell Stem Cell.

[118] Garcez PP, Loiola EC, Madeiro da Costa R, Higa LM, Trindade P, Delvecchio R (2016). Zika virus impairs growth in human neurospheres and brain organoids. Science.

[119] Cugola FR, Fernandes IR, Russo FB, Freitas BC, Dias JL, Guimaraes KP (2016). The Brazilian Zika virus strain causes birth defects in experimental models. Nature.

[120] Andrews MG, Mukhtar T, Eze UC, Simoneau CR, Perez Y, Mostajo-Radji MA, et al. Tropism of SARS-CoV-2 for developing human cortical astrocytes. Natl Acad. Sci. U.S.A. 2022. 10.1073/pnas.2122236119. 10.1073/pnas.2122236119 PMC9335272 35858406

[121] Jacob F, Pather SR, Huang WK, Zhang F, Wong SZH, Zhou H (2020). Human pluripotent stem cell-derived neural cells and brain organoids reveal SARS-CoV-2 neurotropism predominates in choroid plexus epithelium. Cell Stem Cell.

[122] Ramani A, Muller L, Ostermann PN, Gabriel E, Abida-Islam P, Muller-Schiffmann A (2020). SARS-CoV-2 targets neurons of 3D human brain organoids. EMBO J.

[123] Zhang BZ, Chu H, Han S, Shuai H, Deng J, Hu YF (2020). SARS-CoV-2 infects human neural progenitor cells and brain organoids. Cell Res.

[124] Mesci P, de Souza JS, Martin-Sancho L, Macia A, Saleh A, Yin X (2022). SARS-CoV-2 infects human brain organoids causing cell death and loss of synapses that can be rescued by treatment with Sofosbuvir. PLoS Biol.

[125] Seo HH, Han HW, Lee SE, Hong SH, Cho SH, Kim SC (2020). Modelling *Toxoplasma gondii* infection in human cerebral organoids. Emerg Microbes Infect.

[126] Sun G, Chiuppesi F, Chen X, Wang C, Tian E, Nguyen J (2020). Modeling human cytomegalovirus-induced microcephaly in human iPSC-derived brain organoids. Cell Rep. Med.

[127] D’Aiuto L, Bloom DC, Naciri JN, Smith A, Edwards TG, McClain L, et al. Modeling herpes simplex virus 1 infections in human central nervous system neuronal cells using two- and three-dimensional cultures derived from induced pluripotent stem cells. J Virol. 2019;93:e00111-19. 10.1128/JVI.00111-19 PMC6475775 30787148

[128] Dos Reis RS, Sant S, Keeney H, Wagner MCE, Ayyavoo V (2020). Modeling HIV-1 neuropathogenesis using three-dimensional human brain organoids (hBORGs) with HIV-1 infected microglia. Sci Rep.

[129] Bolte S, Girdler S, Marschik PB (2019). The contribution of environmental exposure to the etiology of autism spectrum disorder. Cell Mol Life Sci.

[130] Arzua T, Yan Y, Jiang C, Logan S, Allison RL, Wells C (2020). Modeling alcohol-induced neurotoxicity using human induced pluripotent stem cell-derived three-dimensional cerebral organoids. Transl Psychiatry.

[131] Wang Y, Wang L, Zhu Y, Qin J (2018). Human brain organoid-on-a-chip to model prenatal nicotine exposure. Lab Chip.

[132] Lee CT, Chen J, Kindberg AA, Bendriem RM, Spivak CE, Williams MP (2017). CYP3A5 mediates effects of cocaine on human neocorticogenesis: studies using an in vitro 3D self-organized hPSC model with a single cortex-like unit. Neuropsychopharmacology.

[133] Yin F, Zhu Y, Wang Y, Qin J (2018). Engineering brain organoids to probe impaired neurogenesis induced by cadmium. ACS Biomater Sci Eng.

[134] Cui K, Wang Y, Zhu Y, Tao T, Yin F, Guo Y (2020). Neurodevelopmental impairment induced by prenatal valproic acid exposure shown with the human cortical organoid-on-a-chip model. Microsyst Nanoeng.

[135] Bilinovich SM, Uhl KL, Lewis K, Soehnlen X, Williams M, Vogt D (2020). Integrated RNA sequencing reveals epigenetic impacts of diesel particulate matter exposure in human cerebral organoids. Dev Neurosci.

[136] Zhu Y, Wang L, Yin F, Yu Y, Wang Y, Shepard MJ (2017). Probing impaired neurogenesis in human brain organoids exposed to alcohol. Integr Biol.

[137] Renner H, Becker KJ, Kagermeier TE, Grabos M, Eliat F, Gunther P (2021). Cell-type-specific high throughput toxicity testing in human midbrain organoids. Front Mol Neurosci.

[138] Kim JY, Son MJ, Son CY, Radua J, Eisenhut M, Gressier F (2019). Environmental risk factors and biomarkers for autism spectrum disorder: an umbrella review of the evidence. Lancet Psychiatry.

[139] Cruceanu C, Dony L, Krontira AC, Fischer DS, Roeh S, Di Giaimo R (2022). Cell-type-specific impact of glucocorticoid receptor activation on the developing brain: a cerebral organoid study. Am J Psychiatry.

[140] Pasca AM, Park JY, Shin HW, Qi Q, Revah O, Krasnoff R (2019). Human 3D cellular model of hypoxic brain injury of prematurity. Nat Med.

[141] Lin HY, Perry A, Cocchi L, Roberts JA, Tseng WI, Breakspear M (2019). Development of frontoparietal connectivity predicts longitudinal symptom changes in young people with autism spectrum disorder. Transl Psychiatry.

[142] Oldehinkel M, Mennes M, Marquand A, Charman T, Tillmann J, Ecker C (2019). Altered connectivity between cerebellum, visual, and sensory-motor networks in autism spectrum disorder: results from the EU-AIMS Longitudinal European Autism Project. Biol Psychiatry Cogn Neurosci Neuroimaging.

[143] Redcay E, Courchesne E (2005). When is the brain enlarged in autism? A meta-analysis of all brain size reports. Biol Psychiatry.

[144] Jadav N, Bal VH. Associations between co-occurring conditions and age of autism diagnosis: implications for mental health training and adult autism research. Autism Res. 2022; 10.1002/aur.2808. 10.1002/aur.2808 PMC9637770 36054777

[145] Chau KK, Zhang P, Urresti J, Amar M, Pramod AB, Chen J (2021). Full-length isoform transcriptome of the developing human brain provides further insights into autism. Cell Rep.

[146] Kang HJ, Kawasawa YI, Cheng F, Zhu Y, Xu X, Li M (2011). Spatio-temporal transcriptome of the human brain. Nature.

[147] McDonald-McGinn DM, Sullivan KE, Marino B, Philip N, Swillen A, Vorstman JA (2015). 22q11.2 deletion syndrome. Nat Rev Dis Prim.

[148] Khan TA, Revah O, Gordon A, Yoon SJ, Krawisz AK, Goold C (2020). Neuronal defects in a human cellular model of 22q11.2 deletion syndrome. Nat Med.

[149] Fleck JS, Sanchis-Calleja F, He Z, Santel M, Boyle MJ, Camp JG (2021). Resolving organoid brain region identities by mapping single-cell genomic data to reference atlases. Cell Stem Cell.

[150] Kanton S, Boyle MJ, He Z, Santel M, Weigert A, Sanchis-Calleja F (2019). Organoid single-cell genomic atlas uncovers human-specific features of brain development. Nature.

[151] He Z, Maynard A, Jain A, Gerber T, Petri R, Lin HC (2022). Lineage recording in human cerebral organoids. Nat Methods.

[152] Guan J, Lin Y, Ji G (2020). Cell type-specific gene network-based analysis depicts the heterogeneity of autism spectrum disorder. Front Cell Neurosci.

[153] Velmeshev D, Schirmer L, Jung D, Haeussler M, Perez Y, Mayer S (2019). Single-cell genomics identifies cell type-specific molecular changes in autism. Science.

[154] Maynard KR, Collado-Torres L, Weber LM, Uytingco C, Barry BK, Williams SR (2021). Transcriptome-scale spatial gene expression in the human dorsolateral prefrontal cortex. Nat Neurosci.

[155] Legnini I, Emmenegger L, Zappulo A, Wurmus R, Martinez AO, Jara CC, et al. Spatio-temporal, optogenetic control of gene expression in organoids. [Preprint]. 2021. Available from: 10.1101/2021.09.26.461850.

[156] Genshaft AS, Ziegler CGK, Tzouanas CN, Mead BE, Jaeger AM, Navia AW (2021). Live cell tagging tracking and isolation for spatial transcriptomics using photoactivatable cell dyes. Nat Commun.

[157] Jin S, Guerrero-Juarez CF, Zhang L, Chang I, Ramos R, Kuan CH (2021). Inference and analysis of cell-cell communication using CellChat. Nat Commun.

[158] Hu Y, Sui X, Song F, Li Y, Li K, Chen Z (2021). Lung cancer organoids analyzed on microwell arrays predict drug responses of patients within a week. Nat Commun.

[159] Raghavan S, Winter PS, Navia AW, Williams HL, DenAdel A, Lowder KE (2021). Microenvironment drives cell state, plasticity, and drug response in pancreatic cancer. Cell.

[160] Cadwell CR, Scala F, Li S, Livrizzi G, Shen S, Sandberg R (2017). Multimodal profiling of single-cell morphology, electrophysiology, and gene expression using Patch-seq. Nat Protoc.

[161] Izpisua Belmonte JC, Callaway EM, Caddick SJ, Churchland P, Feng G, Homanics GE (2015). Brains, genes, and primates. Neuron.

[162] Jennings CG, Landman R, Zhou Y, Sharma J, Hyman J, Movshon JA (2016). Opportunities and challenges in modeling human brain disorders in transgenic primates. Nat Neurosci.

[163] Kaiser T, Feng G (2015). Modeling psychiatric disorders for developing effective treatments. Nat Med.

[164] Ilieva M, Aldana BI, Vinten KT, Hohmann S, Woofenden TW, Lukjanska R (2022). Proteomic phenotype of cerebral organoids derived from autism spectrum disorder patients reveal disrupted energy metabolism, cellular components, and biological processes. Mol Psychiatry.

[165] Meng Q, Zhang W, Wang X, Jiao C, Xu S, Liu C (2022). Human forebrain organoids reveal connections between valproic acid exposure and autism risk. Transl Psychiatry.

[166] Rabeling A, Goolam M. Cerebral organoids as an in vitro model to study autism spectrum disorders. Gene Ther. 2022; 10.1038/s41434-022-00356-z. 10.1038/s41434-022-00356-z 35790793

[167] Dixon TA, Muotri AR (2023). Advancing preclinical models of psychiatric disorders with human brain organoid cultures. Mol Psychiatry.

[168] Chen GT, Geschwind DH (2022). Challenges and opportunities for precision medicine in neurodevelopmental disorders. Adv Drug Deliv Rev.

[169] Elamin M, Dumarchey A, Stoddard C, Robinson TM, Cowie C, Gorka D, et al. The role of UBE3A in the autism and epilepsy-related Dup15q syndrome using patient-derived, CRISPR-corrected neurons. Stem Cell Rep. 2023. 10.1016/j.stemcr.2023.02.002. 10.1016/j.stemcr.2023.02.002 PMC10147551 36898382

[170] Foust KD, Nurre E, Montgomery CL, Hernandez A, Chan CM, Kaspar BK (2009). Intravascular AAV9 preferentially targets neonatal neurons and adult astrocytes. Nat Biotechnol.

[171] Meijboom KE, Abdallah A, Fordham NP, Nagase H, Rodriguez T, Kraus C (2022). CRISPR/Cas9-mediated excision of ALS/FTD-causing hexanucleotide repeat expansion in C9ORF72 rescues major disease mechanisms in vivo and in vitro. Nat Commun.

[172] Bowles KR, Silva MC, Whitney K, Bertucci T, Berlind JE, Lai JD (2021). ELAVL4, splicing, and glutamatergic dysfunction precede neuron loss in MAPT mutation cerebral organoids. Cell.

[173] Lange J, Zhou H, McTague A (2022). Cerebral organoids and antisense oligonucleotide therapeutics: challenges and opportunities. Front Mol Neurosci.

[174] Giandomenico SL, Mierau SB, Gibbons GM, Wenger LMD, Masullo L, Sit T (2019). Cerebral organoids at the air-liquid interface generate diverse nerve tracts with functional output. Nat Neurosci.

[175] Krakowiak P, Goines PE, Tancredi DJ, Ashwood P, Hansen RL, Hertz-Picciotto I (2017). Neonatal cytokine profiles associated with autism spectrum disorder. Biol Psychiatry.

[176] Xu ZX, Kim GH, Tan JW, Riso AE, Sun Y, Xu EY (2020). Elevated protein synthesis in microglia causes autism-like synaptic and behavioral aberrations. Nat Commun.

[177] Matsui TK, Tsuru Y, Hasegawa K, Kuwako KI (2021). Vascularization of human brain organoids. Stem Cells.

[178] Sun XY, Ju XC, Li Y, Zeng PM, Wu J, Zhou YY, et al. Generation of vascularized brain organoids to study neurovascular interactions. eLife. 2022;11:e76707. 10.7554/eLife.76707 PMC9246368 35506651

[179] Chico TJA, Kugler EC (2021). Cerebrovascular development: mechanisms and experimental approaches. Cell Mol Life Sci.

[180] Muffat J, Li Y, Omer A, Durbin A, Bosch I, Bakiasi G (2018). Human induced pluripotent stem cell-derived glial cells and neural progenitors display divergent responses to Zika and dengue infections. Proc Natl Acad Sci USA.

[181] Xu R, Boreland AJ, Li X, Erickson C, Jin M, Atkins C (2021). Developing human pluripotent stem cell-based cerebral organoids with a controllable microglia ratio for modeling brain development and pathology. Stem Cell Rep.

[182] Adler R, Canto-Soler MV (2007). Molecular mechanisms of optic vesicle development: complexities, ambiguities and controversies. Dev Biol.

[183] Volkner M, Zschatzsch M, Rostovskaya M, Overall RW, Busskamp V, Anastassiadis K (2016). Retinal organoids from pluripotent stem cells efficiently recapitulate retinogenesis. Stem Cell Rep.

[184] Eiraku M, Takata N, Ishibashi H, Kawada M, Sakakura E, Okuda S (2011). Self-organizing optic-cup morphogenesis in three-dimensional culture. Nature.

[185] Baron-Cohen S, Scott FJ, Allison C, Williams J, Bolton P, Matthews FE (2009). Prevalence of autism-spectrum conditions: UK school-based population study. Br J Psychiatry.

[186] Dakin S, Frith U (2005). Vagaries of visual perception in autism. Neuron.

[187] Fligor CM, Lavekar SS, Harkin J, Shields PK, VanderWall KB, Huang KC (2021). Extension of retinofugal projections in an assembled model of human pluripotent stem cell-derived organoids. Stem Cell Rep.

[188] Leekam SR, Nieto C, Libby SJ, Wing L, Gould J (2007). Describing the sensory abnormalities of children and adults with autism. J Autism Dev Disord.

[189] Mammen MA, Moore GA, Scaramella LV, Reiss D, Ganiban JM, Shaw DS (2015). Infant avoidance during a tactile task predicts autism spectrum behaviors in toddlerhood. Infant Ment Health J.

[190] Orefice LL, Mosko JR, Morency DT, Wells MF, Tasnim A, Mozeika SM (2019). Targeting peripheral somatosensory neurons to improve tactile-related phenotypes in ASD models. Cell.

[191] Orefice LL, Zimmerman AL, Chirila AM, Sleboda SJ, Head JP, Ginty DD (2016). Peripheral mechanosensory neuron dysfunction underlies tactile and behavioral deficits in mouse models of ASDs. Cell.

[192] Wiggins LD, Robins DL, Bakeman R, Adamson LB (2009). Brief report: sensory abnormalities as distinguishing symptoms of autism spectrum disorders in young children. J Autism Dev Disord.

[193] Mazzara PG, Muggeo S, Luoni M, Massimino L, Zaghi M, Valverde PT (2020). Frataxin gene editing rescues Friedreich’s ataxia pathology in dorsal root ganglia organoid-derived sensory neurons. Nat Commun.

[194] Faustino Martins JM, Fischer C, Urzi A, Vidal R, Kunz S, Ruffault PL (2020). Self-organizing 3D human trunk neuromuscular organoids. Cell Stem Cell.

[195] Pereira JD, DuBreuil DM, Devlin AC, Held A, Sapir Y, Berezovski E (2021). Human sensorimotor organoids derived from healthy and amyotrophic lateral sclerosis stem cells form neuromuscular junctions. Nat Commun.

[196] Amadei G, Handford CE, Qiu C, De Jonghe J, Greenfeld H, Tran M (2022). Embryo model completes gastrulation to neurulation and organogenesis. Nature.

[197] Tarazi S, Aguilera-Castrejon A, Joubran C, Ghanem N, Ashouokhi S, Roncato F (2022). Post-gastrulation synthetic embryos generated ex utero from mouse naive ESCs. Cell.

[198] Amin ND, Pasca SP (2022). Mouse embryo models built from stem cells take shape in a dish. Nature.

[199] Farahany NA, Greely HT, Hyman S, Koch C, Grady C, Pasca SP (2018). The ethics of experimenting with human brain tissue. Nature.

[200] George RP, Tollefsen C (2008). Embryo: a defense of human life. J Clin Investig.

[201] Jeziorski J, Brandt R, Evans JH, Campana W, Kalichman M, Thompson E, et al. Brain organoids, consciousness, ethics and moral status. Semin Cell Dev Biol. 2022; 10.1016/j.semcdb.2022.03.020. 10.1016/j.semcdb.2022.03.020 35339359

[202] Lavazza A, Massimini M (2018). Cerebral organoids: ethical issues and consciousness assessment. J Med Ethics.

[203] Revah O, Gore F, Kelley KW, Andersen J, Sakai N, Chen X (2022). Maturation and circuit integration of transplanted human cortical organoids. Nature.

[204] Kapp SK, editor. Autistic community and the neurodiversity movement: stories from the frontline. Singapore: Palgrave Macmillan; 2020.

[205] Cameron L, Murphy J (2007). Obtaining consent to participate in research: the issues involved in including people with a range of learning and communication disabilities. Br J Learn Disabil.

[206] Cascio MA, Weiss JA, Racine E (2021). Making autism research inclusive by attending to intersectionality: a review of the research ethics literature. Rev J Autism Developmental Disord.

[207] Cascio MA, Weiss JA, Racine E, Autism Research Ethics Task Force. Person-oriented ethics for autism research: Creating best practices through engagement with autism and autistic communities. Autism. 2020;24:1676–90. 10.1177/1362361320918763 32551887

[208] Nicolaidis C, Raymaker D, Kapp SK, Baggs A, Ashkenazy E, McDonald K (2019). The AASPIRE practice-based guidelines for the inclusion of autistic adults in research as co-researchers and study participants. Autism.

[209] Meganathan K, Prakasam R, Baldridge D, Gontarz P, Zhang B, Urano F (2021). Altered neuronal physiology, development, and function associated with a common chromosome 15 duplication involving CHRNA7. BMC Biol.

[210] Mihailovich M, Germain P-L, Shyti R, Pozzi D, Noberini R, Liu Y, et al. 7q11.23 CNV alters protein synthesis and REST-mediated neuronal intrinsic excitability. [Preprint]. 2022. Available from: 10.1101/2022.10.10.511483.

[211] Iefremova V, Manikakis G, Krefft O, Jabali A, Weynans K, Wilkens R (2017). An organoid-based model of cortical development identifies non-cell-autonomous defects in Wnt signaling contributing to Miller-Dieker syndrome. Cell Rep.

[212] Cavallo F, Troglio F, Faga G, Fancelli D, Shyti R, Trattaro S (2020). High-throughput screening identifies histone deacetylase inhibitors that modulate GTF2I expression in 7q11.23 microduplication autism spectrum disorder patient-derived cortical neurons. Mol Autism.

[213] Ross PJ, Zhang WB, Mok RSF, Zaslavsky K, Deneault E, D’Abate L (2020). Synaptic dysfunction in human neurons with autism-associated deletions in PTCHD1-AS. Biol Psychiatry.

[214] Wegscheid ML, Anastasaki C, Hartigan KA, Cobb OM, Papke JB, Traber JN (2021). Patient-derived iPSC-cerebral organoid modeling of the 17q11.2 microdeletion syndrome establishes CRLF3 as a critical regulator of neurogenesis. Cell Rep.

[215] Bershteyn M, Nowakowski TJ, Pollen AA, Di Lullo E, Nene A, Wynshaw-Boris A (2017). Human iPSC-derived cerebral organoids model cellular features of lissencephaly and reveal prolonged mitosis of outer radial glia. Cell Stem Cell.

[216] de Jong JO, Llapashtica C, Genestine M, Strauss K, Provenzano F, Sun Y (2021). Cortical overgrowth in a preclinical forebrain organoid model of CNTNAP2-associated autism spectrum disorder. Nat Commun.

[217] Qian X, Su Y, Adam CD, Deutschmann AU, Pather SR, Goldberg EM (2020). Sliced human cortical organoids for modeling distinct cortical layer formation. Cell Stem Cell.

[218] Raj N, McEachin ZT, Harousseau W, Zhou Y, Zhang F, Merritt-Garza ME (2021). Cell-type-specific profiling of human cellular models of fragile X syndrome reveal PI3K-dependent defects in translation and neurogenesis. Cell Rep.

[219] Trujillo CA, Adams JW, Negraes PD, Carromeu C, Tejwani L, Acab A (2021). Pharmacological reversal of synaptic and network pathology in human MECP2-KO neurons and cortical organoids. EMBO Mol Med.

[220] Pigoni M, Uzquiano A, Paulsen B, Kedaigle A, Yang SM, Symvoulidis P, et al. Cell-type specific developmental defects in PTEN-mutant cortical organoids converge on abnormal circuit activity. [Preprint]. 2022. Available from: 10.1101/2022.11.15.516664. 10.1093/hmg/ddad107 PMC10481103 37384417

[221] Paulsen B, Velasco S, Kedaigle AJ, Pigoni M, Quadrato G, Deo AJ (2022). Autism genes converge on asynchronous development of shared neuron classes. Nature.

[222] Papes F, Camargo AP, de Souza JS, Carvalho VMA, Szeto RA, LaMontagne E (2022). Transcription factor 4 loss-of-function is associated with deficits in progenitor proliferation and cortical neuron content. Nat Commun.

[223] Sun AX, Yuan Q, Fukuda M, Yu W, Yan H, Lim GGY (2019). Potassium channel dysfunction in human neuronal models of Angelman syndrome. Science.

[224] Sen D, Voulgaropoulos A, Drobna Z, Keung AJ (2020). Human cerebral organoids reveal early spatiotemporal dynamics and pharmacological responses of UBE3A. Stem Cell Rep.

[225] Villa CE, Cheroni C, Dotter CP, Lopez-Tobon A, Oliveira B, Sacco R (2022). CHD8 haploinsufficiency links autism to transient alterations in excitatory and inhibitory trajectories. Cell Rep.

[226] Wang P, Mokhtari R, Pedrosa E, Kirschenbaum M, Bayrak C, Zheng D (2017). CRISPR/Cas9-mediated heterozygous knockout of the autism gene CHD8 and characterization of its transcriptional networks in cerebral organoids derived from iPS cells. Mol Autism.

[227] Mellios N, Feldman DA, Sheridan SD, Ip JPK, Kwok S, Amoah SK (2018). MeCP2-regulated miRNAs control early human neurogenesis through differential effects on ERK and AKT signaling. Mol Psychiatry.

[228] Zhang W, Ma L, Yang M, Shao Q, Xu J, Lu Z (2020). Cerebral organoid and mouse models reveal a RAB39b-PI3K-mTOR pathway-dependent dysregulation of cortical development leading to macrocephaly/autism phenotypes. Genes Dev.

[229] Wenderski W, Wang L, Krokhotin A, Walsh JJ, Li H, Shoji H (2020). Loss of the neural-specific BAF subunit ACTL6B relieves repression of early response genes and causes recessive autism. Proc Natl Acad Sci USA.

[230] Malara M, Lutz AK, Incearap B, Bauer HF, Cursano S, Volbracht K (2022). SHANK3 deficiency leads to myelin defects in the central and peripheral nervous system. Cell Mol Life Sci.

[231] Wang Y, Chiola S, Yang G, Russell C, Armstrong CJ, Wu Y (2022). Modeling human telencephalic development and autism-associated SHANK3 deficiency using organoids generated from single neural rosettes. Nat Commun.

[232] Mariani J, Coppola G, Zhang P, Abyzov A, Provini L, Tomasini L (2015). FOXG1-dependent dysregulation of GABA/glutamate neuron differentiation in autism spectrum disorders. Cell.

[233] Notaras M, Lodhi A, Barrio-Alonso E, Foord C, Rodrick T, Jones D (2021). Neurodevelopmental signatures of narcotic and neuropsychiatric risk factors in 3D human-derived forebrain organoids. Mol Psychiatry.

[234] Bu Q, Huang Y, Li M, Dai Y, Fang X, Chen K (2020). Acrylamide exposure represses neuronal differentiation, induces cell apoptosis and promotes tau hyperphosphorylation in hESC-derived 3D cerebral organoids. Food Chem Toxicol.

[235] Berdenis van Berlekom A, Kubler R, Hoogeboom JW, Vonk D, Sluijs JA, Pasterkamp RJ, et al. Exposure to the amino acids histidine, lysine, and threonine reduces mTOR activity and affects neurodevelopment in a human cerebral organoid model. Nutrients. 2022;14:2175. 10.3390/nu14102175 PMC9145399 35631316

[236] Huang Y, Dai Y, Li M, Guo L, Cao C, Huang Y (2021). Exposure to cadmium induces neuroinflammation and impairs ciliogenesis in hESC-derived 3D cerebral organoids. Sci Total Environ.

[237] Ao Z, Cai H, Havert DJ, Wu Z, Gong Z, Beggs JM (2020). One-stop microfluidic assembly of human brain organoids to model prenatal cannabis exposure. Anal Chem.

[238] Brown RM, Rana P, Jaeger HK, O’Dowd JM, Balemba OB, Fortunato EA. Human cytomegalovirus compromises development of cerebral organoids. J Virol. 2019;93:e00957-19. 10.1128/JVI.00957-19 PMC6694831 31217239

[239] O’Brien BS, Mokry RL, Schumacher ML, Pulakanti K, Rao S, Terhune SS (2022). Downregulation of neurodevelopmental gene expression in iPSC-derived cerebral organoids upon infection by human cytomegalovirus. iScience.

[240] Sison SL, O’Brien BS, Johnson AJ, Seminary ER, Terhune SS, Ebert AD. Human cytomegalovirus disruption of calcium signaling in neural progenitor cells and organoids. J Virol. 2019;93:e00954-19. 10.1128/JVI.00954-19 PMC6694809 31217241

[241] Yang L, Zou J, Zang Z, Wang L, Du Z, Zhang D (2023). Di-(2-ethylhexyl) phthalate exposure impairs cortical development in hESC-derived cerebral organoids. Sci Total Environ.

[242] Qiao H, Guo M, Shang J, Zhao W, Wang Z, Liu N (2020). Herpes simplex virus type 1 infection leads to neurodevelopmental disorder-associated neuropathological changes. PLoS Pathog.

[243] Qiao H, Zhao W, Guo M, Zhu L, Chen T, Wang J, et al. Cerebral organoids for modeling of HSV-1-induced-amyloid beta associated neuropathology and phenotypic rescue. Int J Mol Sci. 2022;23:5981. 10.3390/ijms23115981 PMC9181143 35682661

[244] Qiao H, Chiu Y, Liang X, Xia S, Ayrapetyan M, Liu S (2023). Microglia innate immune response contributes to the antiviral defense and blood-CSF barrier function in human choroid plexus organoids during HSV-1 infection. J Med Virol.

[245] Zhang X, Lin H, Dong L, Xia Q (2022). Recapitulating influenza virus infection and facilitating antiviral and neuroprotective screening in tractable brain organoids. Theranostics.

[246] Zhang B, He Y, Xu Y, Mo F, Mi T, Shen QS (2018). Differential antiviral immunity to Japanese encephalitis virus in developing cortical organoids. Cell Death Dis.

[247] Adams Y, Clausen AS, Jensen PO, Lager M, Wilhelmsson P, Henningson AJ (2023). 3D blood-brain barrier-organoids as a model for Lyme neuroborreliosis highlighting genospecies dependent organotropism. iScience.

[248] Harbuzariu A, Pitts S, Cespedes JC, Harp KO, Nti A, Shaw AP (2019). Modelling heme-mediated brain injury associated with cerebral malaria in human brain cortical organoids. Sci Rep.

[249] Yao H, Wu W, Cerf I, Zhao HW, Wang J, Negraes PD (2020). Methadone interrupts neural growth and function in human cortical organoids. Stem Cell Res.

[250] Wu W, Yao H, Dwivedi I, Negraes PD, Zhao HW, Wang J (2020). Methadone suppresses neuronal function and maturation in human cortical organoids. Front Neurosci.

[251] Mokry RL, O’Brien BS, Adelman JW, Rosas S, Schumacher ML, Ebert AD (2022). Nitric oxide attenuates human cytomegalovirus infection yet disrupts neural cell differentiation and tissue organization. J Virol.

[252] Cai H, Ao Z, Tian C, Wu Z, Kaurich C, Chen Z (2023). Engineering human spinal microphysiological systems to model opioid-induced tolerance. Bioact Mater.

[253] Kim J, Lee S, Lee J, Park JC, Kim KH, Ko JM (2022). Neurotoxicity of phenylalanine on human iPSC-derived cerebral organoids. Mol Genet Metab.

[254] Harbuzariu A, Nti A, Harp KO, Cespedes JC, Driss A, Stiles JK (2022). Neuregulin-1/ErbB4 signaling modulates Plasmodium falciparum HRP2-induced damage to brain cortical organoids. iScience.

[255] Wang L, Sievert D, Clark AE, Lee S, Federman H, Gastfriend BD (2021). A human three-dimensional neural-perivascular ‘assembloid’ promotes astrocytic development and enables modeling of SARS-CoV-2 neuropathology. Nat Med.

[256] Andrews MG, Mukhtar T, Eze UC, Simoneau CR, Ross J, Parikshak N (2022). Tropism of SARS-CoV-2 for human cortical astrocytes. Proc Natl Acad Sci USA.

[257] Yi SA, Nam KH, Yun J, Gim D, Joe D, Kim YH, et al. Infection of brain organoids and 2D cortical neurons with SARS-CoV-2 pseudovirus. Viruses. 2020;12:1004. 10.3390/v12091004 PMC7551632 32911874

[258] Samudyata, Oliveira AO, Malwade S, Rufino de Sousa N, Goparaju SK, Gracias J (2022). SARS-CoV-2 promotes microglial synapse elimination in human brain organoids. Mol Psychiatry.

[259] McMahon CL, Staples H, Gazi M, Carrion R, Hsieh J (2021). SARS-CoV-2 targets glial cells in human cortical organoids. Stem Cell Rep.

[260] Lee JA, Bae DH, Choi WH, Cho CH, Bang YS, Yoo J (2022). Effects of sevoflurane exposure on fetal brain development using cerebral organoids. J Mol Neurosci.

[261] Zang Z, Yin H, Du Z, Xie R, Yang L, Cai Y (2022). Valproic acid exposure decreases neurogenic potential of outer radial glia in human brain organoids. Front Mol Neurosci.

[262] Watanabe M, Buth JE, Vishlaghi N, de la Torre-Ubieta L, Taxidis J, Khakh BS (2017). Self-organized cerebral organoids with human-specific features predict effective drugs to combat zika virus infection. Cell Rep.

[263] Xu YP, Qiu Y, Zhang B, Chen G, Chen Q, Wang M (2019). Zika virus infection induces RNAi-mediated antiviral immunity in human neural progenitors and brain organoids. Cell Res.

[264] Yoon KJ, Song G, Qian X, Pan J, Xu D, Rho HS (2017). Zika-virus-encoded NS2A disrupts mammalian cortical neurogenesis by degrading adherens junction proteins. Cell Stem Cell.

[265] Janssens S, Schotsaert M, Karnik R, Balasubramaniam V, Dejosez M, Meissner A, et al. Zika virus alters DNA methylation of neural genes in an organoid model of the developing human brain. mSystems. 2018;3:e00219-17. 10.1128/mSystems.00219-17 PMC5801341 29435496

[266] Krenn V, Bosone C, Burkard TR, Spanier J, Kalinke U, Calistri A (2021). Organoid modeling of Zika and herpes simplex virus 1 infections reveals virus-specific responses leading to microcephaly. Cell Stem Cell.

